# Dexmedetomidine ameliorates acute kidney injury by regulating mitochondrial dynamics via the α2-AR/SIRT1/PGC-1α pathway activation in rats

**DOI:** 10.1186/s10020-024-00964-y

**Published:** 2024-10-25

**Authors:** Shuai Zhang, Xiujing Feng, Guiyan Yang, Haoyang Tan, Xin Cheng, Qichao Tang, Haotian Yang, Yuan Zhao, Xuanpan Ding, Siyao Li, Xinyi Dou, Junfeng Li, Huijie Kang, Xingxing Li, Yaxin Ji, Qingdian Hou, Qiuyue An, Hao Fang, Honggang Fan

**Affiliations:** 1 College of Veterinary Medicine, Northeast Agricultural University, Harbin, China; 2 College of Veterinary Medicine, China Agricultural University, Beijing, China; 3 College of Optoelectronic Engineering, Chongqing University, Chongqing, China; 4 College of Animal Science and Technology, Jilin Agricultural Science and Technology University, Jilin, China

**Keywords:** Dexmedetomidine, Acute kidney injury, Apoptosis, Mitochondrial dynamics, α2-AR/SIRT1/PGC-1α

## Abstract

**Background:**

Sepsis-associated acute kidney injury (AKI) is a serious complication of systemic infection with high morbidity and mortality in patients. However, no effective drugs are available for AKI treatment. Dexmedetomidine (DEX) is an alpha 2 adrenal receptor agonist with antioxidant and anti-apoptotic effects. This study aimed to investigate the therapeutic effects of DEX on sepsis-associated AKI and to elucidate the role of mitochondrial dynamics during this process.

**Methods:**

A lipopolysaccharide (LPS)-induced AKI rat model and an NRK-52E cell model were used in the study. This study investigated the effects of DEX on sepsis-associated AKI and the molecular mechanisms using histologic assessment, biochemical analyses, ultrastructural observation, western blotting, immunofluorescence, immunohistochemistry, qRT-PCR, flow cytometry, and si-mRNA transfection.

**Results:**

In rats, the results showed that administration of DEX protected kidney structure and function from LPS-induced septic AKI. In addition, we found that DEX upregulated the α2-AR/SIRT1/PGC-1α pathway, protected mitochondrial structure and function, and decreased oxidative stress and apoptosis compared to the LPS group. In NRK-52E cells, DEX regulated the mitochondrial dynamic balance by preventing intracellular Ca^2+^ overloading and activating CaMKII.

**Conclusions:**

DEX ameliorated septic AKI by reducing oxidative stress and apoptosis in addition to modulating mitochondrial dynamics via upregulation of the α2-AR/SIRT1/PGC-1α pathway. This is a confirmatory study about DEX pre-treatment to ameliorate septic AKI. Our research reveals a novel mechanistic molecular pathway by which DEX provides nephroprotection.

**Supplementary Information:**

The online version contains supplementary material available at 10.1186/s10020-024-00964-y.

## Graphical abstract

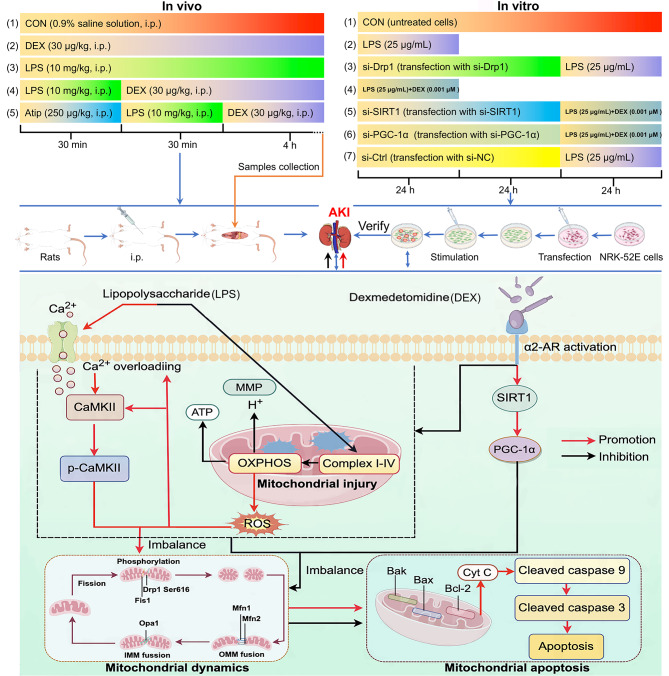

## Introduction

Sepsis is a life-threatening disease in response to extreme pathological stress in the hospital, such as infection, burns, shock, and trauma, posing an important global public health problem (Opal 2010; Singer et al. 2016). Acute kidney injury (AKI) is one of the most common complications of sepsis, occurring in over 50% of patients with sepsis, and the mortality rate is as high as 60% at 3 months (Fan et al. 2022). Therefore, preventing and treating AKI from sepsis is still a major public issue worldwide.

Lipopolysaccharide (LPS) is a major component of the outer membrane of gram-negative bacteria and can cause sepsis-associated AKI by triggering oxidative stress and inflammation (Feng et al. 2019). LPS is widely used for studying sepsis-induced AKI in vitro and in vivo (Qu et al. 2020; Remick et al. 2000). The pathophysiology of sepsis-associated AKI is complex and is characterized by an intense inflammatory response leading to metabolic dysfunction, renal tubule injury, and microvascular dysfunction (Fenhammar et al. 2014; Zarbock et al. 2014). Studies have shown that oxidative stress is important in the primary and secondary injury stages of sepsis-induced AKI (Pavlakou et al. 2017).

Kidney diseases are often recognized as mitochondria-related pathologies (Feng et al. 2022). The energy replenishment required for renal filtration and renal tubule reabsorption depends on mitochondrial integrity. Mitochondria produce adenosine triphosphate (ATP) through oxidative phosphorylation (OXPHOS), playing a key role in cell metabolism. When various stressors damage the mitochondrial respiratory chain complex, electron leakage from complexes I and III increases, leading to excessive reactive oxygen species (ROS) production and accumulation, causing oxidative stress (Apostolova and Victor 2015; Arena et al. 2018). Excessive ROS can enhance the mitochondrial permeability transition pore (mPTP), resulting in mitochondrial DNA damage and reduced mitochondrial membrane potential (MMP) (Rottenberg and Hoek 2017). In kidney diseases, ROS overproduction causes oxidative stress, inducing mitochondrial dysfunction and altering its metabolism and dynamics (Aranda-Rivera et al. 2021). Mitochondrial dysfunction can further increase ROS production and aggravate mitochondrial damage. Numerous studies have demonstrated that AKI is associated with various ultrastructural changes in mitochondria including increases in mitochondrial permeability, MMP reduction, mitochondrial swelling, and ridge rupture (Parikh et al. 2015). Consequently, maintaining mitochondrial homeostasis is essential for preserving kidney health.

Mitochondria are the primary sites of aerobic respiration in cells and are also highly dynamic organelles, undergoing a constant division and fusion, termed mitochondrial dynamics. Mitochondrial fusion including outer mitochondrial membrane (OMM) fusion and inner mitochondrial membrane (IMM) fusion is mediated by mitofusin 1 (Mfn1) and 2 (Mfn 2), regulated by dynamin-like 120 kD protein (Opa1), while division occurs via dynamin-related protein 1 (Drp1) on the outer membrane (Sun et al. 2019; Wang et al. 2022). As dynamic organelles, the balance of mitochondrial fission/fusion is critical for normal function, which is essential for ATP generation, Ca^2+^ homeostasis, and cell survival (Yan et al. 2023). Physiological levels of mitochondrial division are necessary for mitochondrial function, but excessive mitochondrial division/fragmentation can lead to decreased mitochondrial function and increased oxidative stress (Kornfeld et al. 2018). Disruption of mitochondrial dynamics can induce excessive mitophagy and promote apoptosis via increasing oxidative stress. (Li et al. 2019b). However, Mdivi-1, a Drp1 inhibitor, improved mitochondrial function and reduced NLRP3 inflammasome-mediated pyroptosis in tubular epithelial cells in an LPS-induced sepsis-associated AKI model (Liu et al. 2020). Therefore, regulation of mitochondrial dynamics is a potential therapeutic target for LPS-induced sepsis-associated AKI.

Dexmedetomidine (DEX), a highly selective alpha 2 adrenal receptor (α2-AR) agonist, regulates norepinephrine release by acting on presynaptic membrane alpha 2 receptors (Yang et al. 2022). Unlike other sedative drugs, DEX offers superior organ protection with anti-inflammatory, anti-oxidative stress, and anti-apoptotic properties (Hu et al. 2019; Liu et al. 2022; Shi et al. 2021; Sun et al. 2018). DEX protects against diabetes-aggravated brain ischemia-reperfusion (I/R) damage by alleviating oxidative stress, inflammatory response, and apoptosis via the NFAT5/SIRT1/Nrf2 signaling pathway (Apostolova and Victor 2015). In the LPS-induced rat acute lung injury model, DEX reduced the expression of inflammatory cytokines and apoptosis by activating the AMPK/SIRT 1 signaling pathway, thereby alleviating lung injury (Wang et al. 2020). Thus, DEX can play protective roles in organ and tissue injury by regulating sirtuin 1 (SIRT1). SIRT1 deacetylates the transcription factor PGC1α, which, upon deacetylation, engages various binding partners to regulate gene transcription involved in mitochondrial biogenesis and metabolic homeostasis (Patten and Arany 2012). Despite previous studies demonstrating that dexmedetomidine (DEX) reduces organ injury, the role of mitochondrial dynamics, particularly concerning the SIRT1/PGC-1α pathway, has not been investigated as a potential mechanism of nephroprotection with DEX in sepsis-associated acute kidney injury (AKI). Therefore, it remains to be elucidated whether DEX confers protection against sepsis-associated AKI by activating the SIRT1/PGC-1α pathway and regulating mitochondrial function.

Therefore, the study aims to investigate the efficacy of DEX pre-treatment in ameliorating LPS-induced septic AKI in a rat model. We investigated α2-AR/SIRT1/PGC-1α and intracellular Ca^2+^ homeostasis as potential mechanistic pathways in DEX nephroprotection in LPS-induced septic AKI. This is a confirmatory pre-clinical study to evaluate the efficacy of DEX in septic AKI. This study advances understanding by investigating mitochondrial dynamics and calcium homeostasis as mechanistic pathways of DEX nephroprotection.

## Materials and methods

### Animals and experimental design

30 healthy adult male Sprague-Dawley (SD) rats (6 weeks old; weighing 200–240 g) were obtained from the Second Affiliated Hospital of Harbin Medical University (Harbin, Heilongjiang, China). Before experiments, all rats were kept under standard experimental laboratory conditions and a 12 h/12 h light/dark cycle, with free food and water at 21 ± 1 °C for 7 days. This study was approved by the Ethical Committee of Northeast Agricultural University (SRM-11, Harbin, Heilongjiang, China), and experiments were carried out following the institutional guidelines on the care and use of experimental animals.

All rats were randomly divided into five groups (*n* = 6/group): (1) Control group (CON): rats were intraperitoneally injected (i.p.) with 0.9% saline solution; (2) DEX group: rats were treated with DEX (dissolved in 0.9% saline, 30 µg/kg, i.p.); (3) LPS group: rats were injected with LPS (dissolved in 0.9% saline, 10 mg/kg, i.p.); (4) DEX + LPS group: rats were injected with LPS (10 mg/kg, i.p.) 30 min after DEX (30 µg/kg, i.p.) pre-treatment. (5) Atip + DEX + LPS group: rats were pre-treated with the α_2_-AR inhibitor Atipamezole (Atip) (250 µg/kg, i.p.) (Tao et al. 2022). 30 min later, other procedures were consistent with the DEX + LPS group.

### Samples collection

4.5 h after the intraperitoneal injection of LPS, rats were anesthetized with 1.2% isoflurane and 0.5% O_2_, and then urine and blood (5 mL) were collected from the bladder and the heart respectively. Urine and blood samples were left at room temperature for about 30 min, then centrifuged at 3000 rpm for 10 min at 4 °C. Supernatants were used for kidney function tests. Rat kidneys were quickly removed on an ice tray and washed with PBS. The whole left kidney was fixed in 4% paraformaldehyde for hematoxylin-eosin staining (H&E), immunofluorescence (IF), immunohistochemistry (IHC), and TUNEL staining. The right partial fresh kidney was used immediately for ROS and mitochondrial extraction; another part was cut into 1 mm^3^ pieces and fixed in 3% glutaraldehyde for ultrastructural observation. The remaining kidney tissue was immediately frozen in liquid nitrogen and stored at -80 °C for subsequent experiments.

### Cell culture and experimental design

Frozen normal rat kidney-52E (NRK-52E) cells were purchased from Beina Chuang Lianhe Biotechnology Co., LTD. (No.BNCC299115). NRK-52E cells were cultured in DMEM (Gibco, GI, USA) + 10% fetal bovine serum (FBS) + 1% penicillin-streptomycin at 37 °C in an incubator with 5% CO_2_. In vitro, the cultured NRK-52E cells were divided into seven groups: (1) Control group: untreated cells. (2) LPS group: cells were given 25 µg/mL LPS and cultured for 24 h. (3) si-Drp1 + LPS group: cells were stimulated with 25 µg/mL LPS for 24 h after transfection with si-Drp1 for 48 h. (4) DEX + LPS group: cells were stimulated with 25 µg/mL LPS containing 0.001 µM DEX for 24 h. (5) si-SIRT1 + DEX + LPS group: cells were stimulated with 25 µg/mL LPS containing 0.001 µM DEX for 24 h after transfection with si-SIRT1 for 48 h. (6) si-PGC-1α + DEX + LPS group: cells were stimulated with 25 µg/mL LPS containing 0.001 µM DEX for 24 h after transfection with si-PGC-1α for 48 h. (7) si-Ctrl + LPS group: cells were stimulated with 25 µg/mL LPS for 24 h after transfection with si-NC for 48 h.

In vitro, NRK-52E cells were digested with pancreatic enzymes, centrifuged, and resuspended in the fresh medium. They were then inoculated into a 6-well plate, and 2 mL of cell suspension (4 × 10^3^ cells/well) was cultured for 24 h. The si-RNA (si-Drp1 5’ to 3’: F GGGCUAAUGAACAAUAACATT, R UGUUAUUGUUCAUUAGCCCTT; si-SIRT1 5’ to 3’: F CCAGUAGCACUAAUUCCAATT, R UUGGAAUUAGUGCUACUGGTT; si-PGC-1α 5’ to 3’: F CCAAGACUCUAGACAACUATT, R UAGUUGUCUAGAGUCUUGGTT; si-NC) was transfected into the cells, and after 48 h, they were treated with drugs (LPS and/or DEX). The control group was replaced with fresh medium and continued to be cultured for 24 h. Western blot was used for subsequent validation. Then NRK-52E cells were used for subsequent cell experiments.

### Biochemical analyses

Kidney function was assessed by measuring blood urea nitrogen (BUN), serum creatinine (Cre), urinary kidney injury molecule 1 (KIM-1), and urinary neutrophil gelatinase-associated lipocalin (NGAL) levels. Cre and BUN were measured by an AU2700 automatic biochemical analyzer (Olympus, Tokyo, Japan). Urinary NGAL and KIM-1 concentrations were measured using an ELISA kit (Nanjing Jiancheng Bioengineering Institute, Nanjing, China) following the manufacturer’s instructions. Kidney tissues were collected to measure mitochondrial complex I, II, III, IV, glutathione (GSH), glutathione disulfide (GSSG), catalase (CAT), superoxide dismutase (SOD), malondialdehyde (MDA), and ROS to assess oxidative stress level. Levels of complex I, II, III, IV, GSH, GSSG, MDA, and activity of SOD and CAT in kidney tissues were measured using commercially available kits (Nanjing Jiancheng Bioengineering Institute, Nanjing, China) following the manufacturer’s instructions. ROS level was determined using the ROS assay kit (Nanjing Jiancheng Bioengineering Institute, Nanjing, China) (Zhang et al. 2021). Briefly, 50 µg of proteins were added to a 96-well plate and mixed with 100 µL of 2′,7′-dichlorodihydrofluorescein diacetate and then incubated at 37 °C for 30 min in a dark condition. Fluorescence, a positive staining signal (Ex485 nm/Em525 nm) was measured using a Model F4500 fluorescence spectrometer (Hitachi, Tokyo, Japan).

Intracellular Ca^2+^ concentration detection by flow cytometry.

NRK-52E cells were used to measure the intracellular Ca^2+^ content in rats using the annexin Fluo-4 technique. Briefly, the Fluo-4 AM working solution was incubated with NRK-52E cells for 30 min at 37 °C. To confirm that the Fluo-4 AM was completely converted to Fluo-4, they were washed with PBS and incubated for another 20 min. Next, BD FACSAriaTM flow cytometry (BD Biosciences, Beijing, China) was used to assess the Ca^2+^ levels.

### ATP, CaN activity, and MMP detection

Renal and mitochondrial ATP were determined using an assay kit (Nanjing Jiancheng Bioengineering Institute, Nanjing, China) following the manufacturer’s instructions. Fresh kidney tissue was weighed and added with 9 times its volume of boiling double distilled water to make a 10% homogenate. For mitochondrial ATP detection, mitochondria were isolated from kidney tissue using a mitochondrial isolation kit (Nanjing Jiancheng Bioengineering Institute, Nanjing, China). It was then placed in a boiling water bath for 10 min, mixed with a vortex mixer for 1 min at 3500 rpm, and centrifuged for 10 min. The supernatant was used for measurement. The absorbance of each tube was determined at 636 nm. The protein concentration was determined by a bicinchoninic acid (BCA) protein assay kit (Beyotime Biotechnology, Shanghai, China).

In rats, CaN activity and MMP were measured using a calcineurin assay kit (Nanjing Jiancheng Bioengineering Institute, Nanjing, China) and a JC-10 mitochondrial membrane potential detection kit (KeyGEN BioTECH, Jiangsu, China). For MMP detection, NRK-52E cells were incubated in JC-10 liquid for 20 min at 37 °C. Fluorescence (Ex 525 nm/Em 590 nm) was measured using a Model F4500 fluorescence spectrometer (Hitachi, Tokyo, Japan).

### Histologic assessment

The left kidney was fixed in 4% polyformaldehyde for 48 h, embedded in paraffin, and cut into 5-µm sections. The sections were stained with H&E. Images were captured using an Olympus CKX41 microscope (Olympus, Tokyo, Japan) equipped with a Canon EOS 550D camera (Canon, Tokyo, Japan) at a high-magnification field (200 ×).

As previously described, we used a semiquantitative scale score for AKI-associated tubular injury to quantify kidney histological injury in rats (Solanki et al. 2014). Sections were scored by a pathologist unaware of the experimental groups (using 5 random fields, 6 rats/group). Tubular injury is defined by tubular epithelial cell loss, necrosis, tubular epithelial simplification, intratubular debris, and casts. Each parameter was graded as below: 0 = normal, 1 = < 25%, 2 = 25–50%, 3 = 50–75%, 4 = > 75%.

### Ultrastructural observation

The kidney tissue was trimmed into 1 mm^3^ blocks, and fixed with 3% glutaraldehyde for at least 24 h. The tissue was washed three times with phosphate-buffered saline (PBS) for 10 min each, post-fixed with 1% osmium tetroxide for 2 h, and dehydrated in a gradient of alcohols (70%, 80%, 90%, 100% I, 100% II) at 40 °C for 10 min each. It was then embedded in acetone and embedding solution (2:1), followed by acetone and embedding solution (1:2) for 2 h each. The tissue was placed in fresh epoxy resin and polymerized at 60 °C for 2 h. After repairing, ultra-thin Sect. (60 nm) were stained with lead citrate and uranium acetate, and imaged using a transmission electron microscope (Tecnai, Hitachi, Tokyo, Japan) at 100 kV with Electron Microscopy Film 4489 (Kodak, ESTAR thick base, San Francisco, CA).

The mitochondrial morphological damage was graded as below: 0 for no damage (normal mitochondrial morphology), 1 for mild injury (reduced number of cristae and mild swelling of mitochondria), 2 for severe injury (complete loss of cristae and severe mitochondrial swelling), and 3 for destruction (the outer membrane breaks down and the mitochondrial structure collapses) (Stokman et al. 2017). All mitochondria in five separate regions of each sample were scored and the average score for each was plotted. The mean mitochondrial volume was measured using Image-Pro Plus 6.0 software (Media Cybernetics, Washington, USA).

### TUNEL assay

In rats, apoptosis was detected using a TUNEL assay kit (Roche, Basel, Switzerland) according to the manufacturer’s instructions. Images were captured using an Olympus CKX41 microscope (Olympus, Tokyo, Japan) equipped with a Canon EOS 550D camera (Canon, Tokyo, Japan) at a high-magnification field (200 ×).

### Flow cytometry

In vitro, apoptosis was detected using the annexin V-FITC/PI method. Briefly, NRK-52E cells were digested, collected, and centrifuged. The supernatant was discarded. The cells were re-suspended with buffer. Annexin V-FITC was added into the flow tube and the mixture was incubated at room temperature for 15 min in the dark. Then Propyl iodide (PI) and PBS suspensor were added and mixed gently. The apoptosis levels were measured using BD FACSAriaTM flow cytometry (BD Biosciences, Beijing, China).

### Immunohistochemistry

5-µm kidney tissue sections underwent rehydration, and antigen retrieval in 10 mM citrate buffer (pH = 6). They were then treated with 3% hydrogen peroxide (H_2_O_2_) in methanol for 10 min to quench peroxidase activity. After washing, the sections were blocked with 5% goat serum in PBS for 1 h and incubated with primary antibody overnight at 4 °C. The primary antibodies used for IHC were anti-p-CaKII (1:100, Santa Cruz Biotechnology, Texas, USA), anti-SIRT1 (1:100, Santa Cruz Biotechnology, Texas, USA), and anti-PGC-1α (1:200, Wanlei Biotechnology, Shenyang, China). The sections were then incubated with HRP-labeled anti-rabbit IgG (1:500, Zhongshan Golden Bridge Biotechnology Co., Beijing, China) for 15 min at 37 °C. The color reaction of IHC was performed with a coloring kit (Solarbio, Beijing, China) according to the manufacturer’s instructions. Images were captured using an Olympus CKX41 microscope (Olympus, Tokyo, Japan) equipped with a Canon EOS 550D camera (Canon, Tokyo, Japan) at a high-magnification field (200 ×). The IHC quantitative analysis and scoring method were implemented using immunohistochemistry (IHC) Profiler ImageJ software (National Institutes of Health, Bethesda, MD) (Varghese et al. 2014).

### Immunofluorescence

In vivo, 5-µm kidney sections embedded in paraffin were used for IF. After dewaxing, the sections were heated and incubated with 3% H_2_O_2_ for 10 min, followed by blocking with 5% goat serum in PBS for 1 h. The sections were incubated with primary antibodies: anti-Bax (1:300, Wanlei Biotechnology, Shenyang, China) and anti-Tom20 (1:100, ABclonal Technology, Wuhan, China) overnight at 4°C then washed and incubated with fluorescent secondary antibody for 1 h at 37°C. The sections were mounted using 4’,6-diamidino-2‐phenylindole (DAPI) (Good Biotechnology, Co., Ltd., Wuhan, China).

In vitro, NRK-52E cells were fixed with 4% paraformaldehyde and permeabilized with 0.3% Triton X-100. Normal goat serum was added and incubated at room temperature for 30 min. Based on experimental need, cells were incubated with primary antibodies: anti-SIRT1 (1:100, Santa Cruz Biotechnology, Texas, USA), anti-p-Drp1 (Ser616) (1:100, Bioss antibodies, Beijing, China), anti-Mfn2 (1:100, Santa Cruz Biotechnology, Texas, USA), anti-p-CaMKII Thr286 (1:100, Santa Cruz Biotechnology, Texas, USA) overnight at 4 °C followed by incubation with fluorescent secondary antibody for 1 h at 37 °C. If fluorescent double staining was required, the primary antibodies from two species could be added simultaneously, ensuring no antibody cross-reaction. The appropriate fluorescent secondary antibody was selected based on the primary antibody, and its dilution ratio was determined according to the instructions. DAPI drops were added to the cell climbing tablets and then incubated at room temperature for 10 min in the dark. Images were acquired with a Nikon Eclipse Ni inverted microscope (TE2000; Nikon, Tokyo, Japan). IF quantitative analysis was performed using IF Profiler ImageJ software (National Institutes of Health, Bethesda, MD).

### Western blotting.

Samples were lysed in ice-cold RIPA buffer (Sigma-Aldrich, Saint Louis, MO, USA) with protease inhibitor, then centrifuged at 12,000 rpm for 10 min at 4 °C. The supernatants were collected and the total protein concentrations were quantified using a BCA protein assay kit (Beyotime Biotechnology, Shanghai, China). Proteins were separated by sodium dodecyl sulfate-polyacrylamide gel electrophoresis (SDS-PAGE), then blocked in 5% skim milk in TBST (Tris-buffered saline with 0.1% Tween 20) at 37 °C for 2 h. The primary antibodies used were anti-SIRT1 (1:1000, Santa Cruz Biotechnology, Texas, USA), anti-PGC-1α (1:750, Wanlei Biotechnology, Shenyang, China), anti-Drp1 (1:1000, Santa Cruz Biotechnology, Texas, USA), anti-p-Drp1 (Ser616) (1:1000, Bioss antibodies, Beijing, China), anti-p-Drp1 (Ser637) (1:1000, Bioss antibodies, Beijing, China), anti-Fis1 (1:1000, Santa Cruz Biotechnology, Texas, USA), anti-Mfn1 (1:1000, Santa Cruz Biotechnology, Texas, USA), anti-Mfn2 (1:1000, Santa Cruz Biotechnology, Texas, USA), anti-Opa1 (1:1000, Santa Cruz Biotechnology, Texas, USA), anti-Bak (1:1000, Cell Signaling Technology, MA, USA), anti-Bax (1:750, Wanlei Biotechnology, Shenyang, China), anti-Bcl-2 (1:500, Wanlei Biotechnology, Shenyang, China), anti-Cleaved caspases 9 (1:750, Wanlei Biotechnology, Shenyang, China), anti-Cleaved caspases 3 (1:1000, Cell Signaling Technology, MA, USA), anti-Cytochrome C (1:500, Wanlei Biotechnology, Shenyang, China), anti-Pro-caspase 9 (1:750, Wanlei Biotechnology, Shenyang, China), anti-Pro-caspase 3 (1:1000, Santa Cruz Biotechnology, Texas, USA), anti-CaMKII (1:1500, Wanlei Biotechnology, Shenyang, China), anti-p-CaMKII (Thr286) (1:1000, Santa Cruz Biotechnology, Texas, USA), anti-Calcineurin (CaN) (1:1000, Wanlei Biotechnology, Shenyang, China), and anti-GAPDH (1:2000, Cell Signaling Technology, MA, USA). After washing, membranes were incubated with secondary horseradish peroxidase-conjugated antibodies (1:6000, ZSGB-BIO, Beijing, China). Protein bands were visualized using the Tanon 5200 Multi-image system (Tanon Science & Technology Co., Shanghai, China) and were analyzed using Image-Pro Plus 6.0 software (Media Cybernetics, Washington, USA). The results of our study are presented in the form of a ratio of the intensity, which is calculated by dividing the intensity of the target protein bands by the intensity of the GAPDH band.

### Statistics

Statistical analyses were performed by using SPSS Version 25.0 (Chicago, IL, USA). GraphPad Prism Version 10.0 (San Diego, CA, USA) was used for graph generation. All data were analyzed using a one-way analysis of variance (ANOVA) followed by Tukey’s post hoc test. Moreover, the non-parametric Wilcoxon-Mann-Whitney U-test was conducted to compare differences in histologic scores. The integrated optical density and fluorescence intensity were quantitatively analyzed using Image-Pro Plus software (Media Cybernetics, Washington, USA). Image J software was used to analyze the density of protein bands, the average length, and the average volume of mitochondria. Data are expressed as means ± standard error means (SEM). Differences were considered statistically significant at *p* < 0.05 and extremely significant at *p* < 0.01. *p < 0.05, **p < 0.01, *** *p* < 0.001.

## Results

### DEX treatment improved LPS-induced AKI in rats

**Fig. 1:**
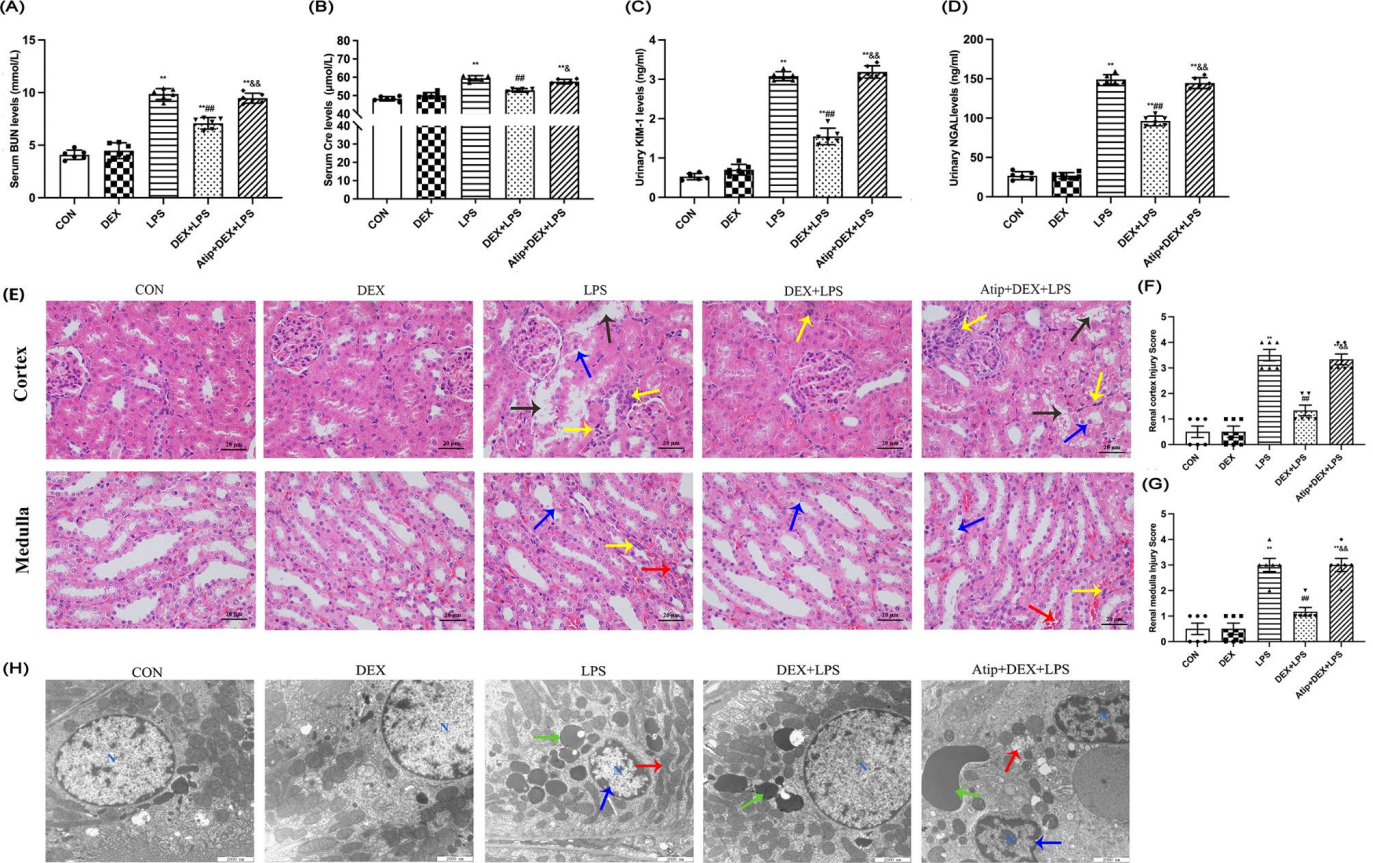
Effect of DEX on LPS-induced AKI. **(A)** Renal BUN level. **(B)** Renal Cre level. **(C)** Urinary KIM-1level. **(D)** Urinary NGAL level. **(E)** Representative kidney histopathological images from each experimental group (400 ×). Black arrows indicated tubular dilatation; yellow arrows indicated inflammatory cell infiltration; blue arrows indicated focal renal tubular necrosis; and red arrows indicated tubular casts. **(F)** Renal cortex injury score. **(G)** Renal medulla injury score. **(H)** Ultrastructural changes of kidney tissue (12000 ×). Blue arrows indicated nuclear damage, red arrows represented mitochondrial damage, and green arrows represented lysosomes. N represents the nucleus. Data are presented as means ± SEM (*n* = 6). ^*^p < 0.05, ^**^p < 0.01 vs. the CON group; ^#^*p* < 0.05, ^##^*p* < 0.01 vs. the LPS group; ^&^*p* < 0.05, ^&&^*p* < 0.01 vs. the DEX + LPS group

**Fig. 2:**
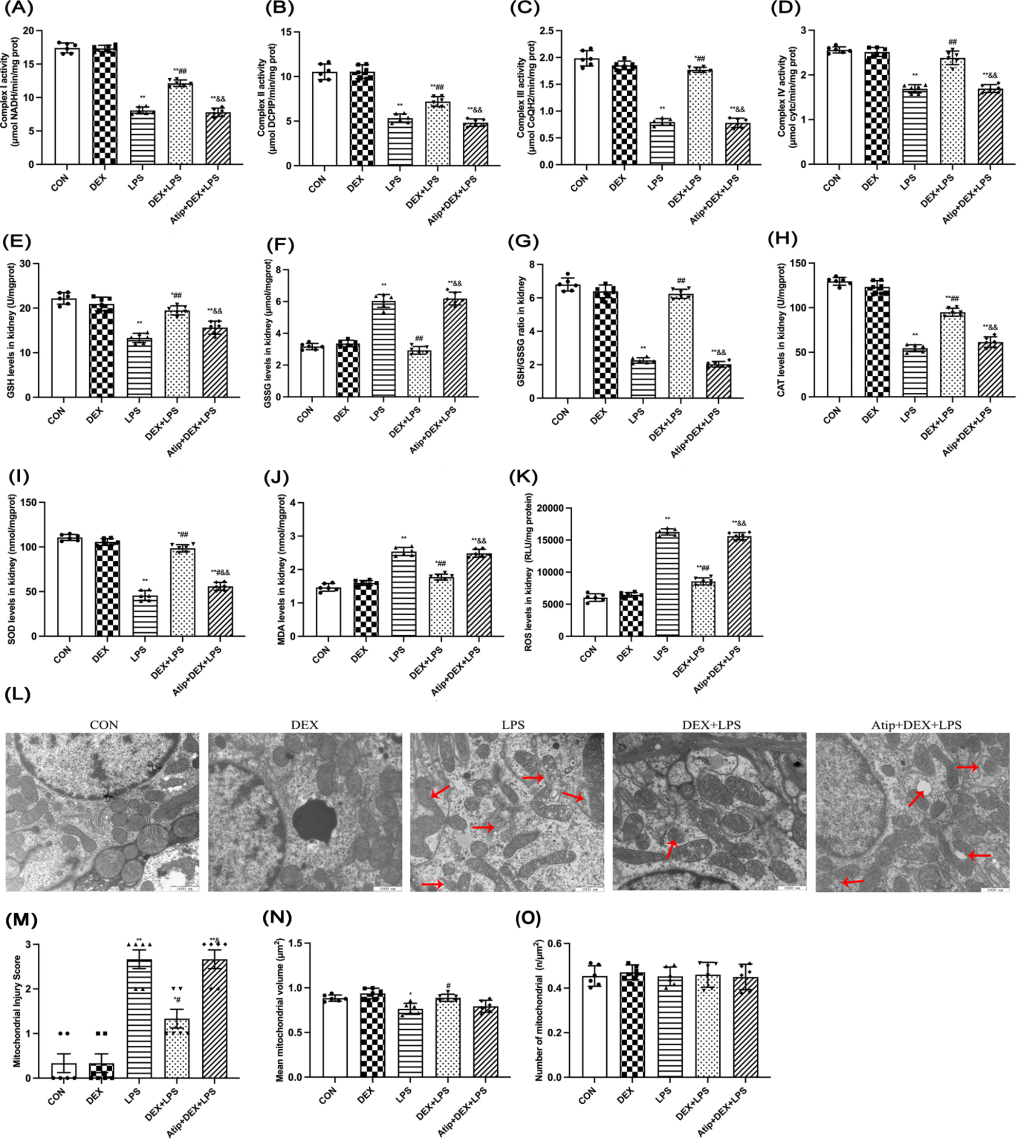
Effect of DEX on mitochondrial injury in the kidney of LPS-induced AKI. Renal **(A)** Complex I, **(B)** Complex II, **(C)** Complex III, **(D)** Complex IV, **(H)** CAT, and **(I)** SOD activity. Renal **(E)** GSH, **(F)** GSSG, **(J)** MDA, and **(K)** ROS Levels. **(L)** Renal ultrastructural mitochondria changes. Red arrows represented mitochondrial damage **(M)** Mitochondrial injury score. **(N)** Mean mitochondrial volume. **(O)** Number of mitochondria. Data are presented as means ± SEM (*n* = 6). ^*^p < 0.05, ^**^p < 0.01 vs. the CON group; ^#^*p* < 0.05, ^##^*p* < 0.01 vs. the LPS group; ^&^*p* < 0.05, ^&&^*p* < 0.01 vs. the DEX + LPS group

BUN, Cre, KIM-1, and NGAL are important indicators for evaluating kidney injury (Fig. 1A-D). Compared with the control (CON) group, BUN, Cre, KIM-1, and NGAL levels in the LPS, DEX + LPS, and Atip + DEX + LPS groups were increased (*p* < 0.01), indicating that LPS-induced AKI in rats (Fig. 1A-D). Compared with the LPS group, BUN, Cre, KIM-1, and NGAL in the DEX + LPS group were decreased (*p* < 0.01), indicating that DEX improved AKI in rats. However, the protective effect of DEX was inhibited by the α2-AR inhibitor Atip administration. Rats in the LPS and Atip + DEX + LPS groups had higher kidney injury scores than those in the CON group (*p* < 0.01; Fig. 1F and G). Compared with the LPS group, the injury score in the DEX + LPS group was lower (*p* < 0.01). The injury score was higher in the Atip + DEX + LPS group compared with the DEX + LPS group (*p* < 0.01; Fig. 1F and G). Electron microscopy (EM) confirmed normal kidney epithelial cell ultrastructure with an intact nuclear (N) and mitochondrial morphology with clearly discernible inner mitochondrial membrane cristae in the CON and DEX groups (Fig. 1H). In contrast, EM examination of tubular epithelial cell ultrastructure of LPS and Atip + DEX + LPS groups showed karyopyknotic nuclear damage, vanishing cristae in swollen mitochondria numerous swollen lysosomes. Similarly, mitochondrial ultrastructural observation showed kidney cells had normal morphology with discernible cristae in CON and DEX rats. Still, mitochondrial damage like swelling, vacuolation, double membrane dissolution, fracture, and blurred ridge structure was observed in the LPS and Atip + DEX + LPS groups. The DEX + LPS group showed only slight mitochondrial swelling (Fig. 2L). The mitochondrial injury score was increased in the LPS, Atip + DEX + LPS groups (*p* < 0.01), and the DEX + LPS group (*p* = 0.0195) compared with the CON group (Fig. 2M). It was decreased in the DEX + LPS group compared with the LPS group (*p* = 0.0153), while it was increased in the Atip + DEX + LPS group compared with the DEX + LPS group (*p* < 0.01). The mean values of mitochondrial volume were decreased in the LPS group compared with the CON group (*p* = 0.0167) and increased in the DEX + LPS group compared with the LPS group (*p* = 0.0147; Fig. 2N). Nonetheless, pretreatment with DEX reduced cell damage, including LPS-induced mitochondrial damage.

### DEX treatment attenuated the LPS-induced oxidative stress during AKI

To assess mitochondrial function, mitochondrial respiratory chain enzyme activity was measured (Fig. 2A-D). Complex I-IV activity was higher in the LPS group compared to the CON group (*p* < 0.01), whereas activity was higher in the DEX group compared to the DEX + LPS group (*p* < 0.01). In addition, the protection effect of DEX was inhibited by Atip administration (*p* < 0.01). Oxidative stress-related indicators including GSH, CAT, SOD, GSSG, MDA, and ROS levels were also measured (Fig. 2E-K). GSH, CAT, and SOD levels decreased in the LPS, DEX + LPS, and Atip + DEX + LPS groups compared with the CON group (*p* < 0.01), and increased in the DEX + LPS compared with the LPS group (*p* < 0.01). However, after Atip administration, GSH, CAT, and SOD levels increased compared with the DEX + LPS group (*p* < 0.01). Compared with the CON group, GSSG, MDA, and ROS levels increased in the LPS and Atip + DEX + LPS groups (*p* < 0.01), and MDA and ROS levels increased in the DEX + LPS group (MDA, *p* = 0.0445; ROS, *p* = 0.0449 ). Additionally, the GSH/GSSG ratio in the LPS and Atip + DEX + LPS groups decreased compared with the CON group (*p* < 0.01) and increased in the DEX + LPS group compared with the LPS group (*p* < 0.01) and decreased in the Atip + DEX + LPS group compared with the DEX + LPS group (*p* < 0.01).

DEX modulated Ca^2+^ transmembrane transport-related factors during LPS-induced AKI.

**Fig. 3:**
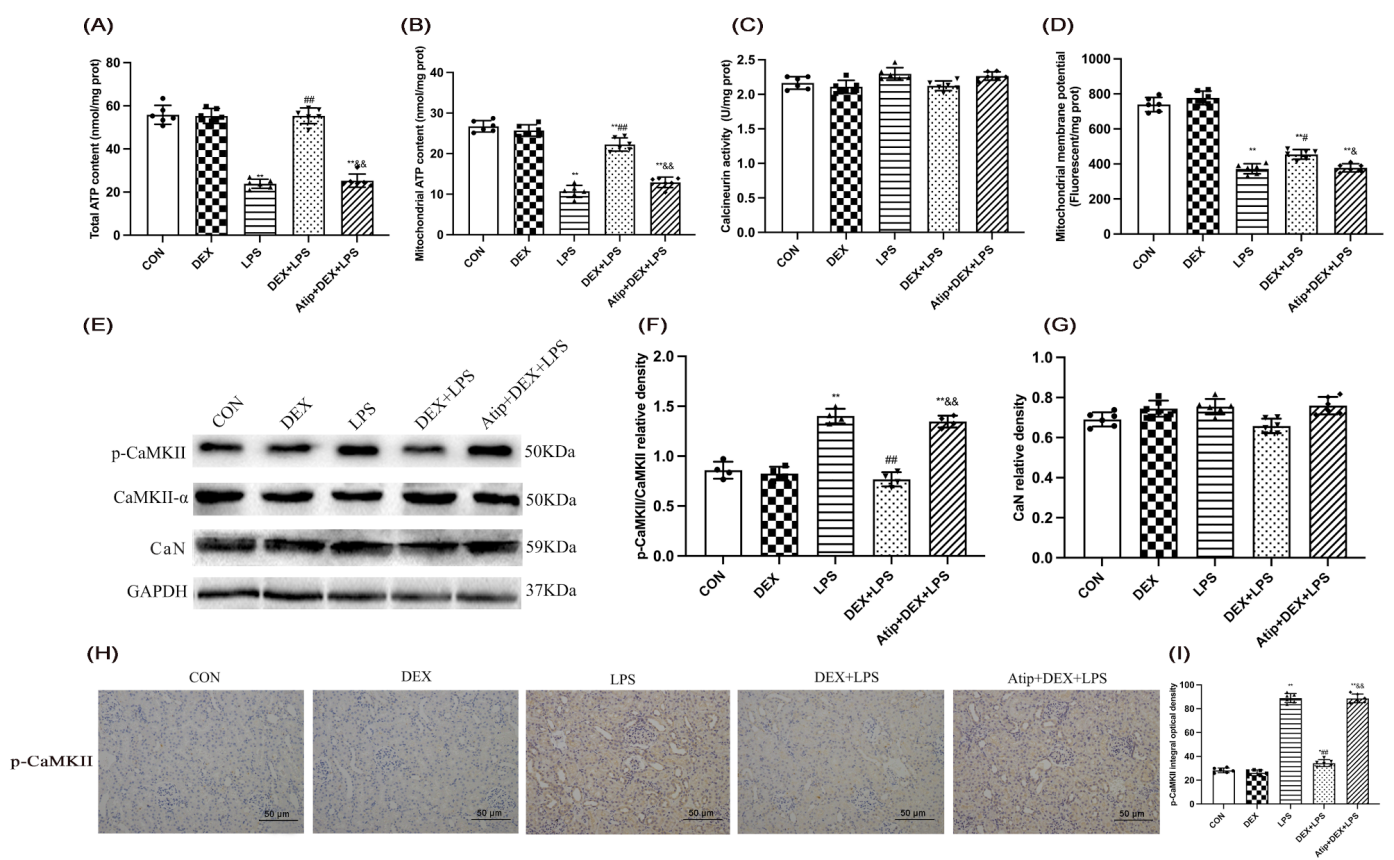
Effect of DEX on Ca^2+^ transmembrane transport-related factors in the kidney of LPS-induced AKI. Renal **(A)** total ATP and **(B)** mitochondrial ATP levels. **(C)** Renal CaN activity. **(D)** Mitochondrial membrane potential. **(E)** Expression of p-CaMKII, CaMKII-α, and CaN proteins. **(F)** Quantitative analysis of p-CaMKII/CaMKII-α. **(G)** Quantitative analysis of CaN protein. Results are presented as the ratio of the intensity of the CaN band to the intensity of the GAPDH band. **(H)** Representative immunohistochemical images of p-CaMKII protein under an optical microscope (200 ×). **(I)** The expression level of the p-CaMKII protein was also evaluated by scoring. Data are presented as means ± SEM (*n* = 6). ^*^p < 0.05, ^**^p < 0.01 vs. the CON group; ^#^*p* < 0.05, ^##^*p* < 0.01 vs. the LPS group; ^&^*p* < 0.05, ^&&^*p* < 0.01 vs. the DEX + LPS group

Ca^2+^ is an essential signaling messenger in regulating various intracellular processes in mitochondria. ATP production and intracellular Ca^2+^ homeostasis play crucial roles in assessing mitochondrial function (Fig. 3). In LPS-induced AKI rats, total and mitochondrial ATP levels were decreased in the LPS and Atip + DEX + LPS groups (*p* < 0.01) and mitochondrial ATP decreased in the DEX + LPS group compared with the CON group (*p* < 0.01; Fig. 3A and B). Total and mitochondrial ATP levels were increased in the DEX + LPS group compared with the LPS group (*p* < 0.01) but decreased in the Atip + DEX + LPS group compared with the DEX + LPS group (*p* < 0.01). The mitochondrial membrane potential (MMP) level was decreased in the LPS, DEX + LPS, and Atip + DEX + LPS groups compared with the CON group (*p* < 0.01), and increased in the DEX + LPS group compared with the LPS group (*p* < 0.01) but decreased in the Atip + DEX + LPS group compared with the DEX + LPS group (*p* = 0.0353, Fig. 3D).

Intracellular Ca^2+^ levels can enhance Ca^2+^-dependent protein kinases (CaMKII) and Calcineurin (CaN). Expression of p-CaMKII, CaMKII-α, and CaN proteins are shown in Fig. 3E. There was an increase in the ratio of p-CaMKII/CaMKII-α in the LPS and Atip + DEX + LPS groups compared with the CON group (*p* < 0.01) and a decrease in the DEX + LPS group compared with the LPS group (*p* < 0.01) and an increase in the Atip + DEX + LPS group compared with the DEX + LPS group (*p* < 0.01; Fig. 3F). There were no differences in CaN activity or protein expression among the five groups (Fig. 3C and G). IHC revealed that the p-CaMKII was mainly localized in renal tubule cells of the kidney (Fig. 3H). The p-CAMKII-positive cells were increased in the LPS, Atip + DEX + LPS, and DEX + LPS groups compared with the CON group (p-CAMKII-positive cells in the LPS group, *p* = 0.0148; p-CAMKII-positive cells in the Atip + DEX + LPS and DEX + LPS groups, *p* < 0.01). The number of positive cells decreased in the DEX + LPS group compared with the LPS group and increased in the Atip + DEX + LPS group compared with the DEX + LPS group (*p* < 0.01). Collectively, these data indicate that DEX modulated Ca^2+^ transmembrane transport-related factors during LPS-induced AKI.

### DEX reversed mitochondrial dynamics via activating the SIRT1/PGC-1α pathway in LPS-induced AKI

**Fig. 4:**
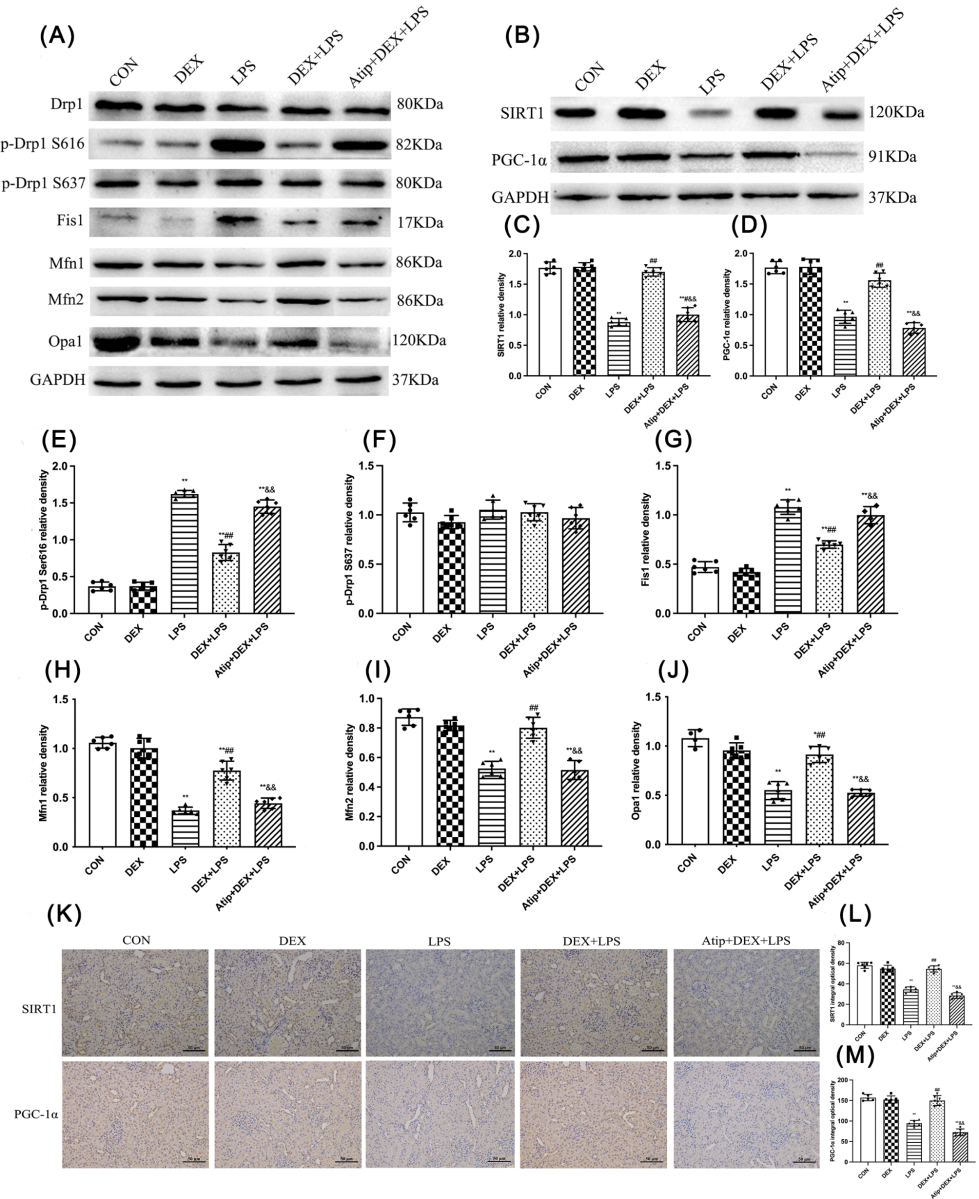
Effect of DEX on the expression of mitochondrial dynamics-related proteins in the kidneys of LPS-induced AKI. **(A)** Renal Drp1, p-Drp1 S616, p-Drp1 S637, Fis1, Mfn1, Mfn2, and Opa1 proteins levels. **(B)** Renal SIRT1, and PGC-1α proteins levels. Quantitative analysis of **(C)** SIRT1, **(D)** PGC-1α, **(E)** p-Drp1 S616, **(F)** p-Drp1 S637, **(G)** Fis1, **(H)** Mfn1, **(I)** Mfn2, and **(J)** Opa1proteins. Results are presented as the ratio of the intensity of the SIRT1, PGC-1α, p-Drp1 S616, p-Drp1 S637, Fis1, Mfn1, Mfn2, and Opa1 bands to the intensity of the GAPDH band respectively. Renal representative immunohistochemical images of SIRT1 and PGC-1α proteins under an optical microscope (200 ×). SIRT1 **(L)** and PGC-1α **(M)** protein levels were evaluated by scoring. Data are presented as means ± SEM (*n* = 6). ^*^p < 0.05, ^**^p < 0.01 vs. the CON group; ^#^*p* < 0.05, ^##^*p* < 0.01 vs. the LPS group; ^&^*p* < 0.05, ^&&^*p* < 0.01 vs. the DEX + LPS group

The expression of the mitochondrial dynamics-related proteins, SIRT1, and PGC1-1α in LPS-induced AKI was detected by western blotting (Fig. 4A and B). Compared with the CON group, p-Drp1 S616 and mitochondrial fission 1 protein (Fis1) expression increased in the LPS, DEX + LPS, and Atip + DEX + LPS groups (*p* < 0.01). Compared with the LPS group, p-Drp1 S616 and Fis1 expression decreased in the DEX + LPS group (*p* < 0.01). Compared with the DEX + LPS group, p-Drp1 S616 and Fis1 expression increased In the Atip + DEX + LPS group (*p* < 0.01; Fig. 4E and G). Compared with the CON group, Mfn1, Mfn2, and Opa1 expression decreased in the LPS and Atip + DEX + LPS groups (*p* < 0.01), and Mnf1 and Opa1 expression decreased in the DEX + LPS group (Mnf1, *p* = 0.0165; Opa1, *p* = 0.0176; Fig. 4H, I, and J). Mfn1, Mfn2, and Opa1 expression increased in the DEX + LPS group compared with the LPS group and decreased in the Atip + DEX + LPS group compared with the DEX + LPS group (*p* < 0.01). SIRT1 and PGC1-1α expression decreased in the LPS and Atip + DEX + LPS groups compared with the CON group (*p* < 0.01). Compared with the LPS group, SIRT1 and PGC1-1α expression increased in the DEX + LPS group (*p* < 0.01) and SIRT1 expression increased in the Atip + DEX + LPS group (*p* = 0.0419). SIRT1 and PGC1-1α expression decreased in the Atip + DEX + LPS group compared with the DEX + LPS group (*p* < 0.01). No differences among groups in p-Drp1 S616 expression were observed (Fig. 4F).

IHC showed that SIRT1 and PGC-1α were highly expressed in kidney tubules (Fig. 4K). IHC and Western blot results showed that the protein expression of SIRT1 and PGC-1α was decreased in the LPS and Atip + DEX + LPS groups compared with the CON group (*p* < 0.01; Fig. 4L and M). It increased in the DEX + LPS group compared with the LPS group (*p* < 0.01) and decreased in the Atip + DEX + LPS group compared with the DEX + LPS group (*p* < 0.01). Thus, these results suggest that DEX reversed mitochondrial dynamics via activating the SIRT1/PGC-1α pathway in LPS-induced AKI.

### DEX treatment inhibited LPS-induced kidney cell apoptosis in rats

**Fig. 5:**
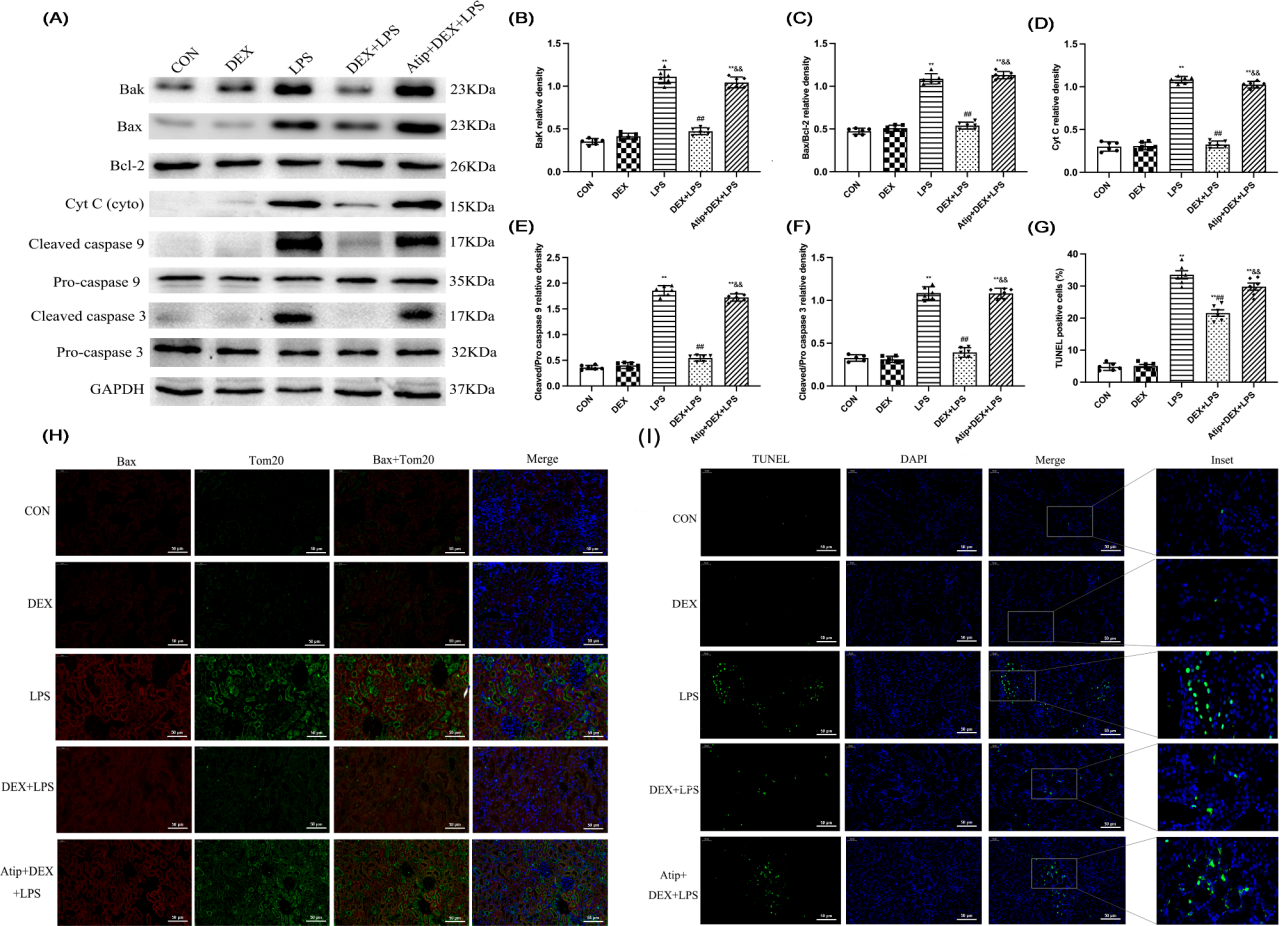
Effect of DEX on the expression of apoptosis-related proteins in the kidneys of LPS-induced AKI. **(A)** Expression of Bak, Cyt C, and Cleaved caspase 9 proteins in the kidney tissues. Results are presented as the ratio of the intensity of the **(B)** Bak, **(D)** Cyt C, and **(F)** Cleaved caspase 9 bands to the intensity of the GAPDH band respectively. **(C)** Quantitative analysis of Bax/Bcl-2 in the kidney tissues. **(F)** Quantitative analysis of Cleaved/Pro caspase 3 in the kidney tissues. **(H)** Colocalization of Bax and mitochondria. Positive Bax and Tom20 were stained red and green respectively, with the sections counterstained with DAPI to visualize nuclei. Merge is a combination of Bax, Tom20, and DAPI. **(I)** TUNEL detected apoptosis. **(G)** TUNEL-positive cells level. Data are presented as means ± SEM (*n* = 6). ^*^p < 0.05, ^**^p < 0.01 vs. the CON group; ^#^*p* < 0.05, ^##^*p* < 0.01 vs. the LPS group; ^&^*p* < 0.05, ^&&^*p* < 0.01 vs. the DEX + LPS group

Mitochondrial dysfunction can induce cell apoptosis. The protein expression of apoptosis-related factors (Bak, Bax/Bcl-2, Cyt C, Cleaved/Pro caspase 9, and Cleaved/Pro caspase 3) was all increased in the LPS and Atip + DEX + LPS groups compared with the CON group (*p* < 0.01; Fig. 5A-F). These proteins were decreased in the DEX + LPS group compared with the LPS group and increased in the Atip + DEX + LPS group compared with the DEX + LPS group (*p* < 0.01; Fig. 5A-F).

Bax activation leads to mitochondrial outer membrane insertion, causing mitochondrial sensitivity and the release of apoptotic factors like Cyt C. The co-localization of Bax and mitochondria was detected through immunofluorescence (Fig. 5H). In the LPS group, Bax (red fluorescence) and Tom20 (green fluorescence) displayed strong fluorescence intensity and higher co-localization than the other groups, mainly in renal tubule cells. The fluorescence intensity and co-localization of Bax and Tom20 were stronger and higher in the Atip + DEX + LPS group compared with the DEX + LPS group.

Additionally, the transferase-mediated deoxyuridine triphosphate-biotin nick end labeling (TUNEL) assay revealed that apoptosis (red fluorescence) in the kidney was induced in all rats injected with LPS in comparison with CON and DEX groups (Fig. 5I, G). Apoptosis was increased in the Atip + DEX + LPS group compared with the DEX + LPS group. These results indicate that LPS promoted renal tubule cell apoptosis, and DEX reversed this effect by activating α2-AR.

### DEX alleviated LPS-induced cell damage in NRK-52E cells

**Fig. 6:**
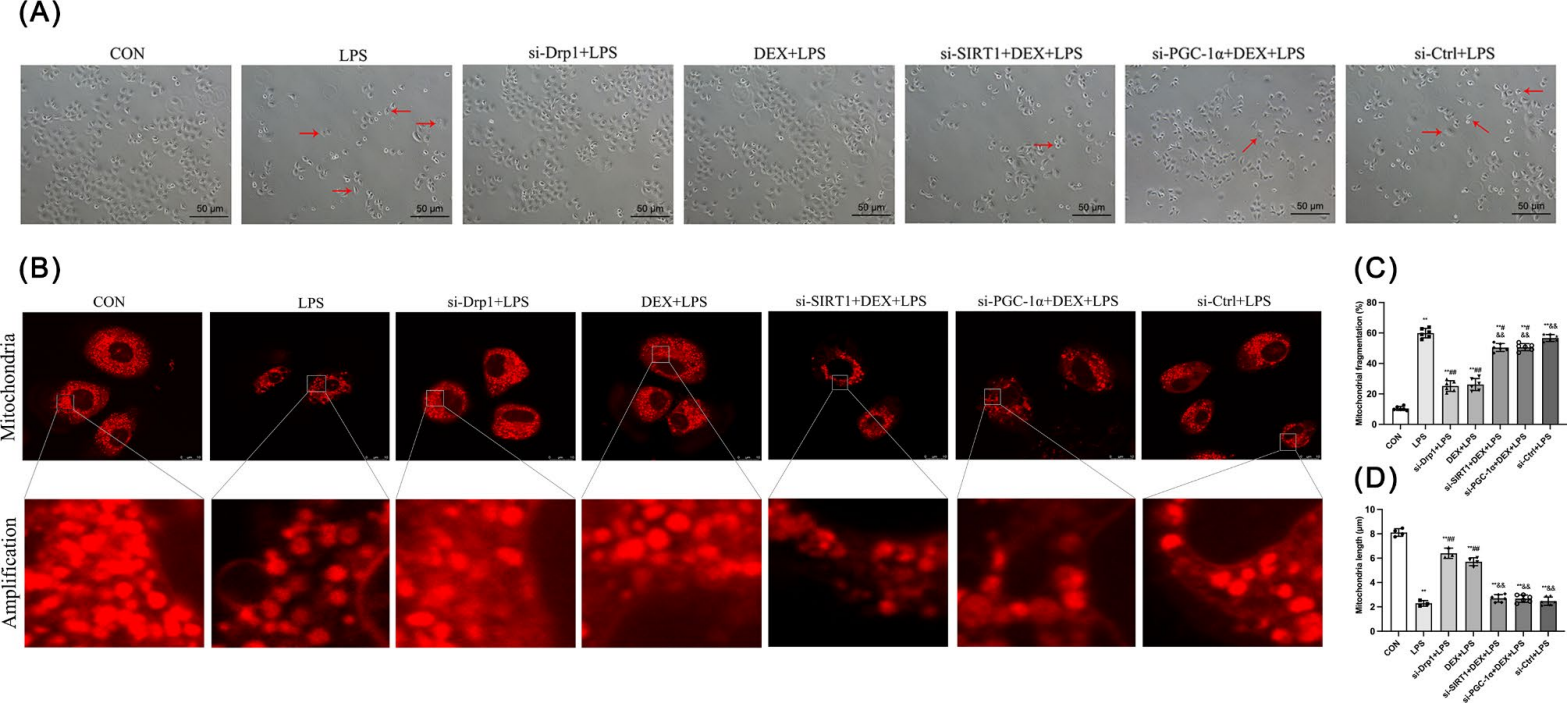
Effect of DEX on oxidative stress-induced NRK-52E cell injury and mitochondrial injury in NRK-52E cells. **(A)** Cell morphology was observed in the difference group (20 × 10). **(B)** Mitochondrial morphology was observed by laser confocal microscopy. **(C)** The mitochondrial fragmentation level. **(D)** Mitochondria length. Data are presented as means ± SEM (*n* = 6). ^*^p < 0.05, ^**^p < 0.01 vs. the CON group; ^#^*p* < 0.05, ^##^*p* < 0.01 vs. the LPS group; ^&^*p* < 0.05, ^&&^*p* < 0.01 vs. the DEX + LPS group

In NRK-52E cells, normal CON group displayed good growth and uniformity (Fig. 6A). After overgrowth, cells developed a pebble-like growth pattern with tight connections. LPS infected cells showed abnormal morphology, poor growth, and necrosis (red arrows). Cells in the si-Drp1 + LPS and DEX + LPS groups were normal with some loose connections and a few necrotic cells. Cells in the si-SIRT1 + DEX + LPS, si-PGC-1α + DEX + LPS, and si-Ctrl + LPS groups were similar to the LPS group, with poor growth and weak connections (red arrows). These data indicate protective effects of DEX against LPS-induced cytotoxicity.

DEX ameliorated the LPS-induced mitochondrial morphological abnormalities (disintegrating the tubular network structure and reducing branching) in NRK-52E cells (Fig. 6B). These cell changes were associated with enhancing mitochondrial network structure and reducing fragmentation. As expected, si-Drp1 and si-SIRT1 interfered with DEX’s effect to improve mitochondrial morphology during LPS infection.

Mitochondrial length and fragmentation analysis were also studied (Fig. 6C and D). Compared with the CON group, the other six groups showed increased mitochondrial fragmentation and reduced mitochondrial length (*p* < 0.01). Compared with the LPS group, si-Drp1 + LPS and DEX + LPS groups had decreased fragmentation and increased mitochondrial length (*p* < 0.01), and DEX-treated LPS infection with si-SIRT1 or si-PGC-1α exhibited decreased fragmentation in NRK-52E cells. si-SIRT1 or si-PGC-1α led to a higher fragmentation degree and shorter mitochondrial length than in the DEX + LPS group without inhibition (*p* < 0.01). There were no significant differences in fragmentation degree and mitochondrial length between the LPS and si-Ctrl + LPS groups. Collectively, these results suggest that both si-Drp1 and DEX prohibited LPS-induced mitochondrial fragmentation, and this protective effect of DEX is dependent on SIRT1 and PGC-1α activation.

### DEX alleviated LPS-induced oxidative stress in NRK-52E cells

**Fig. 7:**
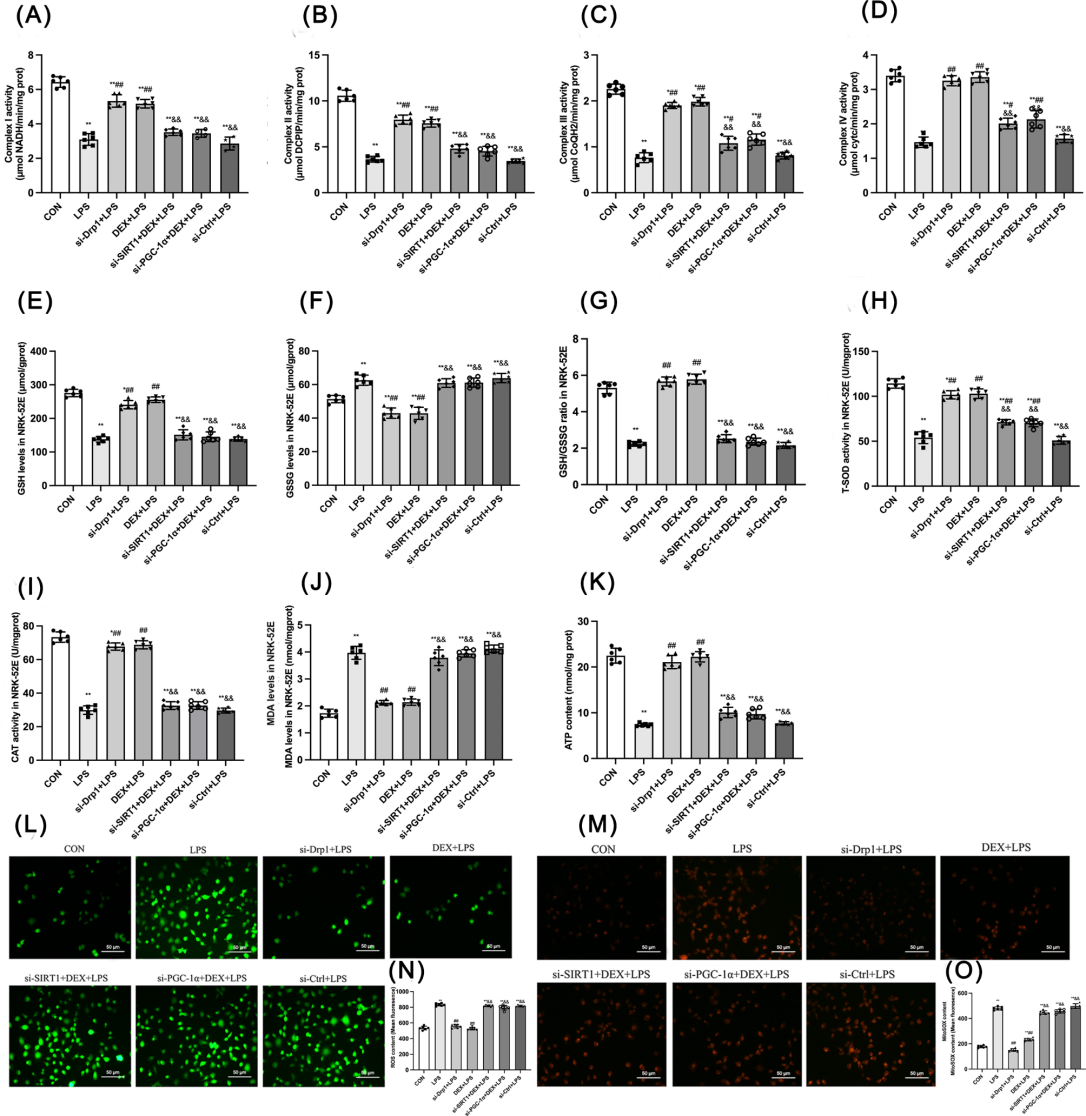
Effect of DEX on oxidative stress-related factors in NRK-52E cells. The activity of **(A)** Complex I, **(B)** Complex II, **(C)** Complex III, **(D)** Complex IV, **(H)** T-SOD, and **(I)** CAT. The **(E)** GSH, **(F)** GSSG, **(J)** MDA, and **(K)** ATP levels. **(G)** The ratio of GSH/GSSG. **(L)** Representative images of ROS under an optical microscope (200 ×). **(M)** Representative images of mitochondrial ROS under an optical microscope (200 ×). **(N)** The ROS level. **(O)** The mitochondrial ROS level. Data are presented as means ± SEM (*n* = 6). ^*^p < 0.05, ^**^p < 0.01 vs. the CON group; ^#^*p* < 0.05, ^##^*p* < 0.01 vs. the LPS group; ^&^*p* < 0.05, ^&&^*p* < 0.01 vs. the DEX + LPS group

The mitochondrial respiratory chain complexes I-IV activity was tested (Fig. 7A-D). Compared with the CON group, complex I, II, III, and IV activities were decreased in all the other groups (*p* < 0.01), while these activity changes were abolished by si-Drp1 or DEX in response to LPS (*p* < 0.01). Compared with the DEX + LPS group, complex I-IV activity was decreased by si-SIRT1 and si-PGC-1α (*p* < 0.01). Compared with the LPS group, complex III and IV activity were increased by si-Drp1 and DEX (*p* < 0.01).

For oxidative stress markers (Fig. 7D-J), T-SOD, CAT, GSH, GSH/GSSG, and ATP content were decreased, whereas GSSG and MDA were increased in LPS groups compared with the control (*p* < 0.01). Compared with the LPS group, T-SOD, CAT, GSH, GSH/GSSG, and ATP content were increased while GSSG and MDA were decreased by si-Drp1 and DEX (*p* < 0.01). Compared with the DEX + LPS group, T-SOD, CAT, GSH, GSH/GSSG, and ATP content were decreased, while GSSG and MDA were increased by si-SIRT1 and si-PGC-1α (*p* < 0.01).

Intracellular and mitochondrial ROS in NRK-52E cells were further detected (Fig. 7L-O). Compared with the CON group, intracellular and mitochondrial ROS was increased in the LPS groups (*p* < 0.01). Compared with the LPS group, it was decreased after si-Drp1 and DEX treatment (*p* < 0.01). However, this effect of DEX was blocked by si-SIRT1 and si-PGC-1α (*p* < 0.01). Taken together, LPS caused oxidative stress, ROS production, and cytotoxicity. si-Drp1 and DEX treatments could reduce oxidative stress and enhance mitochondrial function via SIRT1 and PGC-1α activation.

DEX alleviated LPS-induced cell damage via regulating Ca^2+^ transmembrane transport and associated factors.

**Fig. 8:**
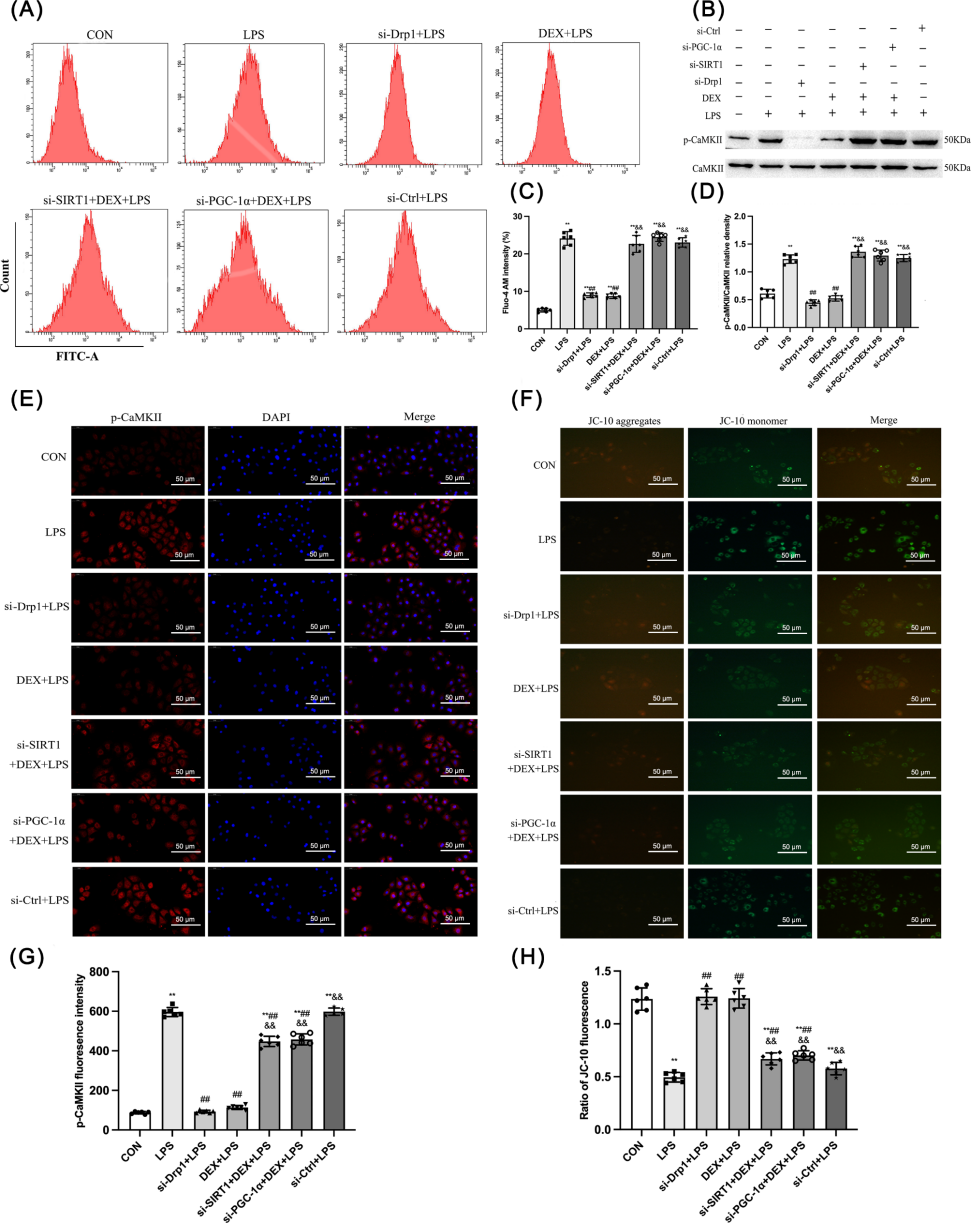
Effect of DEX on Ca^2+^ transmembrane transport and related factors in LPS-induced NRK-52E cell damage. **(A)** Ratio of p-CaMKII/CaMKII-α. **(B)** Detection of intracellular Ca^2+^ levels by Flow cytometry. **(C)** Quantitative analysis of p-CaMKII/CaMKII-α. **(D)** Fluo-4 AM intensity. **(E)** Expression of p-CaMKII by immunofluorescence (200 ×). **(F)** Results of MMP in NRK-52E cells (200 ×). **(G)** p-CaMKII fluorescence intensity. **(H)** Ratio of JC-10 fluorescence. Data are presented as means ± SEM (*n* = 6). ^*^p < 0.05, ^**^p < 0.01 vs. the CON group; ^#^*p* < 0.05, ^##^*p* < 0.01 vs. the LPS group; ^&^*p* < 0.05, ^&&^*p* < 0.01 vs. the DEX + LPS group

Ca^2+^ is a versatile signal conductor mediating numerous cellular responses. Disruption of Ca^2+^ homeostasis plays a critical role in cell damage and death. In NRK-52E cells, flow cytometry results showed that the Ca^2+^ concentration was elevated by LPS compared with the CON group (*p* < 0.01; Fig. 8A and C), and this increase was inhibited by si-Drp1 and DEX (*p* < 0.01). si-SIRT1, si-PGC-1α, or si-Ctrl-treated groups still had higher Ca^2+^ levels than the cells only treated with DEX or si-Drp1 in response to LPS (Fig. 8A and C).

Furthermore, p-CaMKII and CaMKII protein expression in NRK-52E cells was detected by Western blotting (Fig. 8B and D). Consistently, the ratio of p-CaMKII/CaMKII was elevated by LPS compared with the CON group (*p* < 0.01), and this decrease was abolished by si-Drp1 and DEX treatment (*p* < 0.01). However, the total protein expression of CaMKII was not changed among groups. Moreover, immunofluorescence results revealed that si-Drp1 and DEX prevented the LPS-increased p-CaMKII expression (*p* < 0.01; Fig. 8E). si-SIRT1 and si-PGC-1α inhibited DEX-reduced p-CaMKII expression in response to LPS (Fig. 8B, D, G).

In addition, the MMP decline is a key event of mitochondrial dysfunction and the start of the apoptotic cascade. The MMP decrease can cause an increase in mitochondrial membrane permeability, leading to the release of mitochondrial apoptosis factors into the cytoplasm. The MMP fluorescence of the CON group was yellow-green (Fig. 8F). High MMP results in aggregation of JC-10 in the mitochondrial matrix, emitting red fluorescence, while low MMP leads to JC-10 emitting green fluorescence. Quantitative analysis showed that MMP level was decreased by LPS compared with the CON group (*p* < 0.01), this reduction was prevented by si-Drp1 and DEX. However, the effect of si-Drp1 and DEX on upregulating MMP was abolished by si-SIRT1 or si-PGC-1α (Fig. 8F, H). Thus, DEX is capable of maintaining Ca^2+^ homeostasis and MMP via SIRT1 and PGC-1α.

DEX reversed mitochondrial dynamics via activating the SIRT1/PGC-1α pathway in NRK-52E cells in response to LPS.

**Fig. 9:**
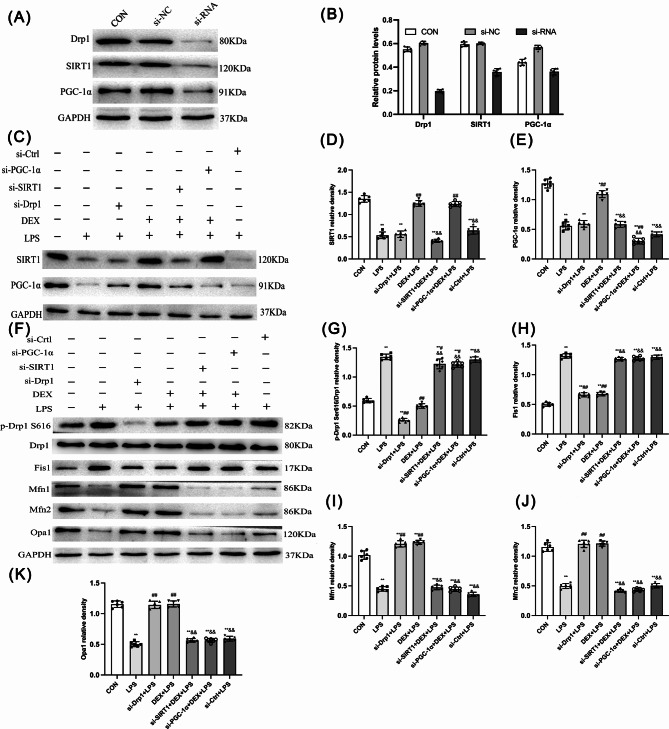
Effect of DEX on the expression of mitochondrial dynamics-related proteins in NRK-52E cells. **(A)** The expression levels of Drp1, SIRT1, and PGC-1α proteins after si-NC and si-RNA administration. **(B)** Quantitative analysis of Drp1, SIRT1, and PGC-1α proteins after si-NC and si-RNA administration. **(C)** The SIRT1 and PGC-1α proteins levels. Quantitative analysis of **(D)** SIRT1 and **(E)** PGC-1αproteins. **(F)** The p-Drp1 S616, Drp1, Fis l, Mfn 1, Mfn 2, and Opal 1 proteins levels in different groups. **(G)** The ratio of p-Drp1 S616/Drp1. Quantitative analysis of **(H)** Fis l, **(I)** Mfn 1, **(J)** Mfn 2, and **(K)** Opal 1 proteins. Data are presented as means ± SEM (*n* = 6). ^*^p < 0.05, ^**^p < 0.01 vs. the CON group; ^#^*p* < 0.05, ^##^*p* < 0.01 vs. the LPS group; ^&^*p* < 0.05, ^&&^*p* < 0.01 vs. the DEX + LPS group; ^+^*p* < 0.05, ^++^*p* < 0.01, vs. the si-Drp1 + LPS group; ^ *p* < 0.05, ^^ *p* < 0.01 vs. the si-SIRT1 + DEX + LPS group

Samples were collected 48 h post-transfection of si-Drp1, si-SIRT1, si-PGC-1α, and si-NC into NRK-52E cells (Fig. 9A). Compared with control and si-NC groups, protein expressions of Drp1, SIRT1, and PGC-1α were decreased (*p* < 0.01) after si-Drp1, si-SIRT1, and si-PGC-1α transfection, indicating Drp1, SIRT1, and PGC-1α were successfully silenced (Fig. 9B).

Western blot results showed that mitochondrial dynamics-related proteins (SIRT1 and PGC-1α) expression was decreased by LPS or si-Drp1 compared with the control in NRK-52E cells (Fig. 9C-E). p-Drp1/Drp1 and Fis1 levels were decreased after si-Drp1 and DEX treatment in response to LPS (*p* < 0.01; Fig. 9F-H). si-SIRT1 and si-PGC-1α inhibited DEX-reduced p-Drp1/Drp1 and Fis1 expression in response to LPS (Fig. 9F-H). s.

LPS decreased the fusion proteins (Mfn1, Mfn2, and Opa1) compared with the CON group (*p* < 0.01). However, Mfn1, Mfn2, and Opa1 levels were increased after si-Drp1 and DEX treatment in response to LPS (*p* < 0.01; Fig. 9F, I-K). si-SIRT1 and si-PGC-1α inhibited these DEX-elevated fusion proteins’ expression in response to LPS (*p* < 0.01; Fig. 9F, I-K).

**Fig. 10:**
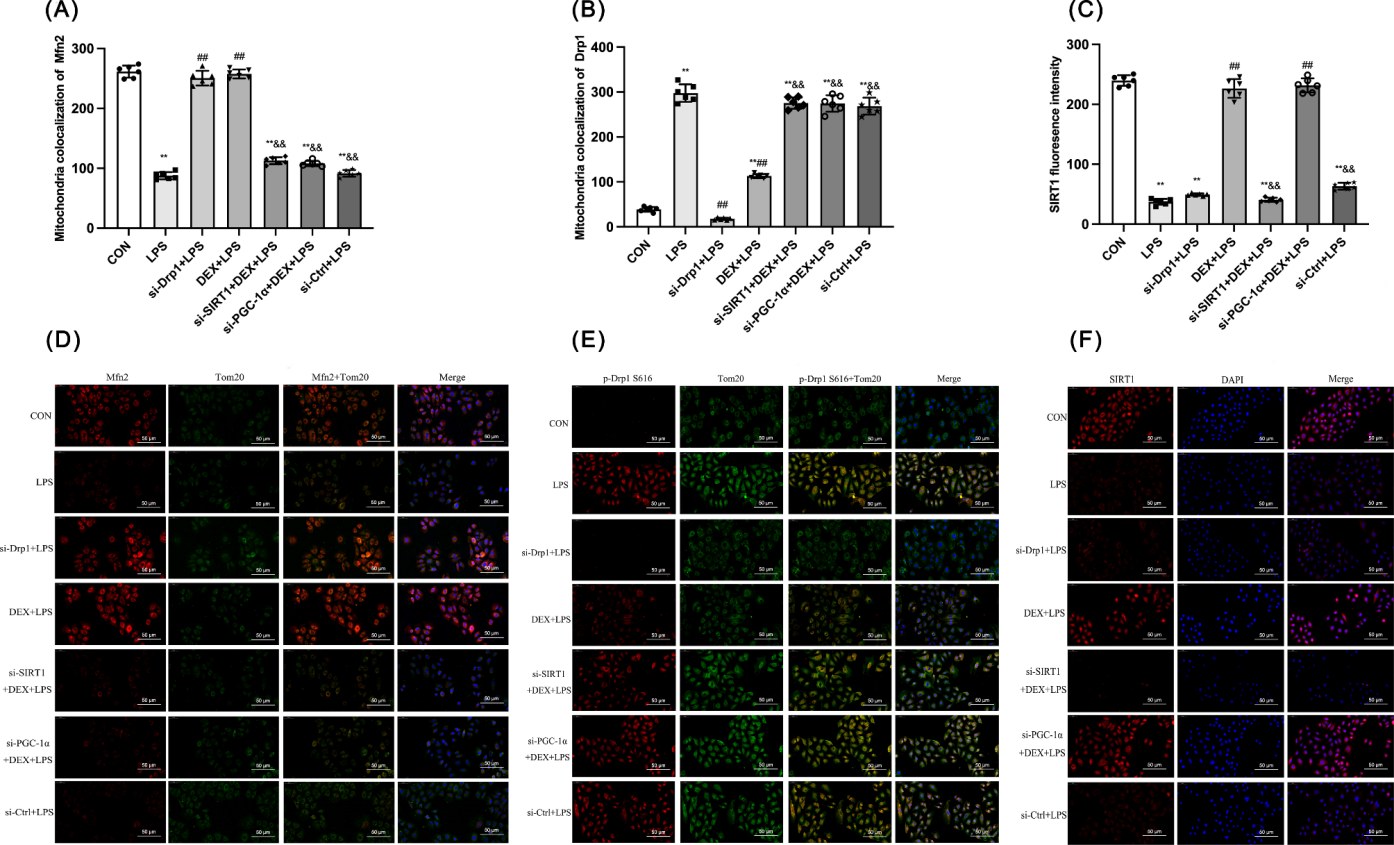
Effect of DEX on the expression of mitochondrial dynamics-related proteins in NRK-52E cells. Quantitative analysis of **(A)** mitochondrial Mfn2 and **(B)** mitochondrial p-Drp1 S616. **(C)** SIRT1 fluorescence intensity. **(D)** Colocalization of Mfn2 and mitochondria. **(E)** Colocalization of p-Drp1 S616 and mitochondria. Positive Mfn2 and Tom20 were stained red and green respectively, with the sections counterstained with DAPI to visualize nuclei. Merge is a combination of Mfn2, Tom20, and DAPI. **(F)** Expression of SIRT1 by immunofluorescence (200 ×). Data are presented as means ± SEM (*n* = 6). ^*^p < 0.05, ^**^p < 0.01 vs. the CON group; ^#^*p* < 0.05, ^##^*p* < 0.01 vs. the LPS group; ^&^*p* < 0.05, ^&&^*p* < 0.01 vs. the DEX + LPS group; ^+^*p* < 0.05, ^++^*p* < 0.01, vs. the si-Drp1 + LPS group

Immunofluorescence further assessed the role of Mfn2 and Drp1 in regulating mitochondrial dynamics (Fig. 10D and E). Mfn2 and Tom20 co-localization was decreased by LPS compared with the CON group (*p* < 0.01), indicating LPS inhibited mitochondrial fusion (Fig. 10A). si-Drp1 and DEX could increase the LPS-induced co-localization of Mfn2 and Tom20 (*p* < 0.01), whereas this effect of DEX was abolished after silencing SIRT1 and PGC-1α (*p* < 0.01; Fig. 10A). In contrast, p-Drp1 s616 and Tom20 co-localization was increased by LPS compared with the CON group (*p* < 0.01) and was decreased by Drp1 silencing + and DEX treatment compared with the LPS group (*p* < 0.01). Silencing SIRT1 and PGC-1α increased p-Drp1 s616 positive signal in DEX-treated LPS-infected cells (*p* < 0.01; Fig. 10B). No significant difference in the Mfn2/Tom20 and p-Drp1 S616/Tom20 co-localization was observed between the LPS and si-Ctrl + LPS groups.

SIRT1 fluorescence intensity was decreased in the LPS, si-Drp1 + LPS, si-SIRT1 + DEX + LPS, and si-Ctrl + LPS groups compared with the CON group (*p* < 0.01). DEX treatment or/and PGC-1α silence increased SIRT1 in response to LPS (*p* < 0.01). Therefore, DEX upregulated the SIRT1 and PGC-1α signaling pathways independent of the *Drp1* gene. DEX reversed mitochondrial dynamics via activating the SIRT1/PGC-1α pathway, contributing to alleviating LPS-induced NRK-52E cell damage.

### DEX inhibited LPS-induced NRK-52E cell apoptosis via SIRT1/PGC-1α

**Fig. 11:**
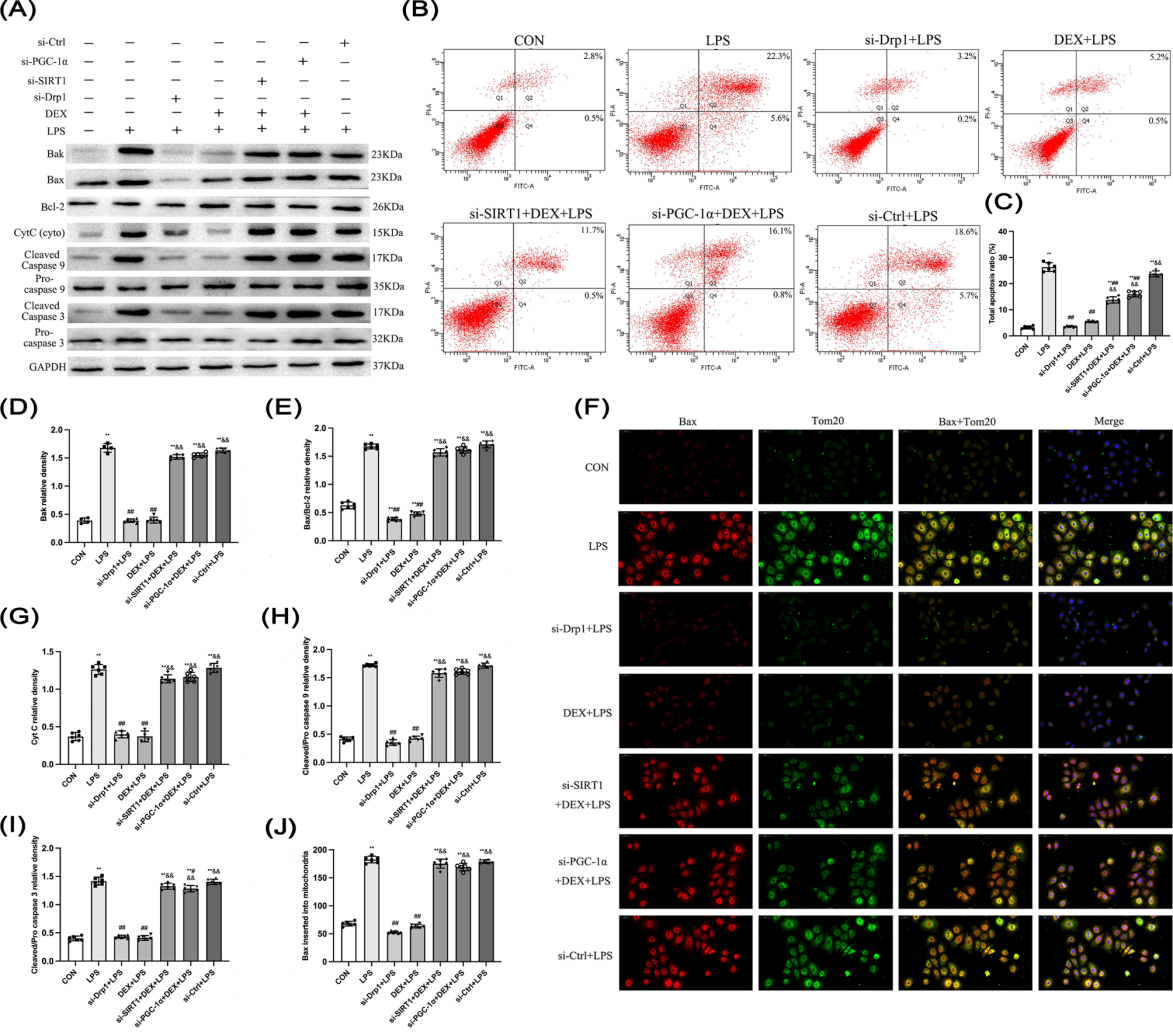
Effect of DEX on the expression of apoptosis-related proteins in NRK-52E cells. **(B)** The level of apoptosis was detected by flow cytometry. **(C)** The ratio of total apoptosis. Quantitative analysis of **(D)** Bak and **(G)** Cyt C proteins. The ratio of **(E)** Bax/Bcl-2, **(H)** Cleaved/Pro Caspase 9, and **(I)** Cleaved/Pro Caspase 3. **(F)** Colocalization of Bax and mitochondria (200 ×). **(J)** Quantitative analysis of Bax inserted into mitochondria. Bax-positive and Tom20-positive cells are labeled fluorescently red and green respectively. The yellow in the merged images represents the degree of colocalization between Drp1 (red) and mito (green). Data are presented as means ± SEM (*n* = 6). ^*^p < 0.05, ^**^p < 0.01 vs. the CON group; ^#^*p* < 0.05, ^##^*p* < 0.01 vs. the LPS group; ^&^*p* < 0.05, ^&&^*p* < 0.01 vs. the DEX + LPS group

Western blotting and flow cytometry showed that Bak, Bax/Bcl-2, Cyt C, Cleaved/Pro caspase 9, and Cleaved/Pro caspase 3 expression as well as the apoptosis rate were increased by LPS compared with the CON group, indicating LPS induced NRK-52E cell apoptosis (Fig. 11). Silencing Drp1 and DEX treatment reduced the expression of proteins involved in apoptosis and apoptosis rate induced by LPS, whereas this phenomenon was not observed after silencing SIRT1 and PGC-1α. Simultaneously, immunofluorescence was used to detect Bax and Tom20 co-localization (Fig. 11F). Consistent with the Western blot results, Bax and Tom20 co-localization was increased by LPS, and reduced by si-Drp1 and DEX during LPS challenge dependent of SIRT1 and PGC-1α. Thus, both Drp1 silence and DEX reduced NRK-52E cell apoptosis, and the anti-apoptotic effect of DEX is partly dependent on SIRT1 and PGC-1α activation.

## Discussion

AKI associated with sepsis is a serious complication of systemic infection with high morbidity and mortality. In this study, an LPS-induced AKI rat model and NRK-52E cells were used to investigate the role of mitochondrial dynamics in kidney disease progression, especially during DEX treatment. AKI caused by sepsis often shows sudden renal dysfunction and histopathological changes (Bagshaw et al. 2008; Heyman et al. 2002). Increased BUN, Cre, NGAL, and KIM-1 indicated LPS-induced renal dysfunction in the LPS group. Histologic and ultrastructural observation also showed more serious pathological changes in the LPS group. Thus, LPS successfully induced sepsis-associated AKI in rats. DEX pre-treatment provided structural and functional kidney protection as shown by KIM1, ultrastructural, histological assessment, and BUN, Cre, and NGAL measurements respectively. Furthermore, Atip exacerbated renal failure and pathological alterations in the rat AKI model while inhibiting the protective effects of DEX. Our findings suggest that DEX mainly used its binding to α2-AR to protect the kidneys.

LPS is a known bacterial toxin that can induce oxidative stress in various pathological models (Yan et al. 2023; Zhang et al. 2022; Zhou et al. 2021). Oxidative stress damage plays a key role in the pathogenesis of AKI (Chen et al. 2018). Our study showed that LPS disrupted the antioxidant defense system and caused oxidative stress damage in rats and NRK-52E cells. Additionally, LPS impaired the normal mitochondrial respiratory chain function by inhibiting complex I-IV activity to induce mitochondrial dysfunction and reduce ATP production. Similarly, the results of mitochondrial ultrastructure confirmed that oxidative stress damage occurred in the mitochondria. Simultaneously, the mitochondrial injury-induced MMP changes reflected underlying mitochondrial dysfunction as well. After DEX pre-treatment, oxidative stress injuries in rats improved by promoting the complex I-IV activity, reducing the oxidative stress level, and reversing MMP and ATP production in kidney tissue and NRK-52E cells. These findings suggest that LPS-induced AKI can be prevented by DEX due to lowering oxidative stress generated by mitochondrial dysfunction and structural damage.

Under physiological conditions, mitochondrial dynamics ensures efficient mitochondrial OXPHOS and ATP production (Punter et al. 2023). During metabolic or environmental stress, mitochondrial homeostasis shifts to division, accompanied by morphological fragmentation, impaired energy metabolism, increased ROS, and loss of MMP (Baker et al. 2019; Serasinghe et al. 2015). Mitochondrial division mediates dysfunction and induces AKI under various pathological conditions (Funk and Schnellmann 2013; Yuan et al. 2018). Drp1, typically in the cytoplasm, was translocated to the MOM upon activation and bound Fis1, triggering mitochondrial division (Loson et al. 2013). Drp1 transcription, expression, and mitochondrial translocation are important factors in regulating its function (Ding et al. 2018). In the present study, the experiment used immunofluorescence triple staining to determine if Drp1 translocation to Fis1 at the division site in the MOM mediates LPS-induced AKI. Our study found that LPS promoted p-Drp1Ser616 expression and Drp1 translocation to MOM where it bound Fis1 while decreasing the Mfn1/2 and Opa1 expression, promoting mitochondrial division and disrupting mitochondrial division fusion balance. DEX pretreatment inhibited Drp1 mitochondrial translocation, reducing its binding with Fis1, and suppressing mitochondrial division while Atip markedly reversed DEX’s regulation of mitochondrial dynamics. This suggested mitochondrial dynamic imbalance as a key in LPS-induced AKI. DEX pre-treatment inhibited Drp1mitochondrial translocation, reduced Drp1-Fis1 binding, inhibited the mitochondrial division, and promoted fusion through α2-AR. However, Atip reversed the DEX’s regulatory effect on mitochondrial dynamics. Overall analysis suggests a new potential protective mechanism of DEX on LPS-induced AKI by regulating the mitochondrial dynamic balance.

Disruption of Ca^2+^ homeostasis leads to changes in mitochondrial structure and function (Verdejo et al. 2012). Elevated intracellular Ca^2+^ can regulate CaN activation and phosphorylation to upregulate Drp1, accelerating its migration to mitochondria and stimulating division (Pennanen et al. 2014). Increased Ca^2+^ also can enhance CaMKII, directly phosphorylating Drp1 at Ser616, causing mitochondrial breakage (Yang et al. 2021). Our study showed that LPS only induced CaMKII activation and increased p-CaMKII expression in kidney cortex tubules, leading to Drp1 Ser616 phosphorylation and promoting mitochondrial division. Previous studies have shown that ROS can enhance CaMKII, leading to phosphorylation at Thr286 of CaMKII (Jain et al. 2014; Premkumar and Chaube 2016). Thus, both Ca^2+^ overload and ROS elevation promote LPS-induced cellular CaMKII activation, suggesting LPS-induced oxidative stress promotes cellular events leading to Ca^2+^ influx, playing a role in LPS-induced mitochondrial dysfunction. CaMKII can be a key component in the Ca^2+^ and ROS-mediated signaling cascade, leading to LPS-induced mitochondrial breakdown. LPS treatment induces oxidative stress and Ca^2+^ overload in NRK-52E cells, leading to Drp1 phosphorylation at Ser616, promoting Drp1 shift to mitochondria and excessive mitochondrial division. While silencing Drp1 with small interfering RNA inhibited LPS-induced Ca^2+^ influx and activation of CaMKII in NRK-52E cells. DEX pre-treatment can reduce p-CaMKII levels and alleviate ROS-mediated Ca^2+^ overload, inhibiting phosphorylation of Drp1 Ser616 and its translocation to mitochondria caused by CaMKII activation, thereby inhibiting mitochondrial division and protecting AKI caused by sepsis while Atip reversed this down-regulation. Similarly, DEX prevents remifentanil-induced postoperative hyperalgesia by modulating the NMDAR-PKC-Ca^2+^/CaMKII pathway in the spinal cord (Yuan et al. 2017). Therefore, DEX may inhibit Ca^2+^-associated CaMKII increase through α2-AR, inhibiting mitochondrial division in the LPS-induced AKI.

Respiratory chain dysfunction by excessive ROS can damage mitochondrial DNA, enhance mitochondrial division, and promote the release of apoptotic proteins (Baker et al. 2019). Studies have shown excessive mitochondrial division leads to apoptosis of renal tubular cells during AKI (Liu et al. 2019). Cyt C is released from MOM simultaneously with mitochondrial fragmentation during apoptosis. Drp1-dependent mitochondrial division regulates Cyt C release through mitochondrial DNA distribution and ridge remodeling (Ban-Ishihara et al. 2013). Drp1 may interact with Bax to initiate mitochondrial fission, changing morphology and permeability (Wei et al. 2013), leading to excessive mitochondrial division and apoptosis. In rat AKI models and NRK-52E cells, inhibition of Bax or Drp1 protects mitochondrial integrity and function, reducing apoptosis and renal tubule damage. Additionally, this experiment also found that silencing Drp1 with small interfering RNA inhibited mitochondrial division, alleviated LPS-induced apoptosis of NRK-52E cells, blocked Cyt C release, and prevented apoptosis induced by mitochondrial division. Cell fluorescence double staining showed colocalization of Bax and mitochondria were significantly inhibited, MMP stabilized, and mitochondrial morphology restored after silencing Drp1. The silencing Drp1 effects were consistent with DEX treatment, significantly reducing apoptosis and inhibiting mitochondrial translocation of Bax. Thus, DEX may play a protective role in LPS-induced apoptosis of mitochondrial pathway cells by inhibiting mitochondrial division.

SIRT1 is a key oxidative stress suppressor and has protective effects on kidney injury in various models (Funk et al. 2010; Li et al. 2019a; Yoshizaki et al. 2010). PGC-1α is a transcription factor and key downstream target of SIRT1, regulating mitochondrial biotransformation and metabolism. In our study, DEX pretreatment increased SIRT1 and PGC-1α expression in the tubular cells of the renal cortex in LPS-induced AKI. However, it’s unclear if DEX-induced upregulation of SIRT1/PGC-1α expression is related to its regulation of mitochondrial dynamics. In this study, silencing SIRT1 with small interfering RNA in vitro, DEX blocked the inhibition of mitochondrial division induced by LPS-induced AKI and the promotion of mitochondrial fusion, as shown by increased expression of fission protein (Drp1 Ser616 and Fis1) and decreased expression of fusion protein (Mfn1/2 and Opa1). These results suggest that the regulation of DEX on mitochondrial dynamics may depend on the role of SIRT1. Studies have reported that a SIRT1 inhibitor was used to demonstrate SIRT1’s key role in DEX protection functions, including alleviating postoperative cognitive dysfunction in older rats (Fang et al. 2018). Application of α2-AR inhibitor Atip reversed the activation of DEX on the SIRT1/PGC-1α pathway. Thus, the protective effect of DEX on LPS-induced AKI in rats may be closely related to the activation of the α2-AR/SIRT1/PGC-1α signaling pathway.

In conclusion, DEX ameliorates AKI by reducing oxidative stress and apoptosis, which regulates mitochondrial dynamics via activating the α2-AR/SIRT1/PGC-1α pathway. This is a confirmatory study about DEX pre-treatment to ameliorate septic AKI. Our research reveals a novel mechanistic molecular pathway by which DEX provides nephroprotection.

## Electronic supplementary material

Below is the link to the electronic supplementary material.

Supplementary Material 1

## Data Availability

No datasets were generated or analysed during the current study.

## Abbreviations

AKI: acute kidney injury
AMPK: adenosine 5’-monophosphate (AMP)-activated protein kinase
ANOVA: one-way analysis of variance
Atip: atipamezole
ATP: adenosine triphosphate
Bak: Bcl-2 homologous antagonist/killer
BCA: bicinchoninic acid
Bcl-2: B-cell lymphoma 2
BUN: blood urea nitrogen
CaMKII: Ca^2+^-dependent protein kinases
CaN: calcineurin
CAT: catalase
Cre: serum creatinine
Cyt C: cytochrome c
DAPI: 4,6-diamino-2-phenylindole
DEX: dexmedetomidine
Drp1: dynamin-related protein 1
EM: electron microscopy
Fis1: mitochondrial fission 1 protein
GAPDH: glyceraldehyde-3-phosphate dehydrogenase
GSH: glutathione
GSSG: glutathione disulfide
H&E: hematoxylin-eosin staining
H_2_O_2_: hydrogen peroxide
I/R: ischemia-reperfusion
ICU: intensive care unit
IF: immunofluorescence
IHC: immunohistochemistry
IMM: inner mitochondrial membrane
KIM-1: kidney injury molecule 1
LPS: lipopolysaccharide
MDA: malondialdehyde
Mfn1: mitofusin 1
Mfn2: mitofusin 2
MMP: mitochondrial membrane potential
mPTP: mitochondrial permeability transition pore
NFAT5: nuclear factor of activated T cells 5
NGAL: neutrophil gelatinase-associated lipocalin
Nrf2: nuclear factor erythroid 2-related factor 2
NRK-52E: normal rat kidney-52E
OMM: outer mitochondrial membrane
Opa1: dynamin-like 120 kD protein
OXPHOS: oxidative phosphorylation
PGC-1α: peroxisome proliferator-activated receptorγcoactivator-1α
PI: propyl iodide
RNA: ribonucleic acid
ROS: reactive oxygen species
SD: Sprague-Dawley
SDS-PAGE: sodium dodecyl sulfate-polyacrylamide gel electrophoresis
SEM: standard error means
SIRT1: Sirtuin 1
SOD: superoxide dismutase
TBST: Tris-buffered saline with 0.1% Tween 20
TUNEL: transferase-mediated deoxyuridine triphosphate-biotin nick end labeling
α2-AR: alpha 2 adrenal receptor
